# Using a Bayesian network to understand the importance of coastal storms and undeveloped landscapes for the creation and maintenance of early successional habitat

**DOI:** 10.1371/journal.pone.0209986

**Published:** 2019-07-25

**Authors:** Sara L. Zeigler, Benjamin T. Gutierrez, Emily J. Sturdivant, Daniel H. Catlin, James D. Fraser, Anne Hecht, Sarah M. Karpanty, Nathaniel G. Plant, E. Robert Thieler

**Affiliations:** 1 U.S. Geological Survey, Woods Hole Coastal and Marine Science Center, Woods Hole, Massachusetts, United States of America; 2 Department of Fish and Wildlife Conservation, Virginia Tech, Blacksburg, Virginia, United States of America; 3 U.S. Fish and Wildlife Service, Sudbury, Massachusetts, United States of America; 4 U.S. Geological Survey, St. Petersburg Coastal and Marine Science Center, St. Petersburg, Florida, United States of America; Feroze Gandhi Degree College, INDIA

## Abstract

Coastal storms have consequences for human lives and infrastructure but also create important early successional habitats for myriad species. For example, storm-induced overwash creates nesting habitat for shorebirds like piping plovers (*Charadrius melodus*). We examined how piping plover habitat extent and location changed on barrier islands in New York, New Jersey, and Virginia after Hurricane Sandy made landfall following the 2012 breeding season. We modeled nesting habitat using a nest presence/absence dataset that included characterizations of coastal morphology and vegetation. Using a Bayesian network, we predicted nesting habitat for each study site for the years 2010/2011, 2012, and 2014/2015 based on remotely sensed spatial datasets (e.g., lidar, orthophotos). We found that Hurricane Sandy increased piping plover habitat by 9 to 300% at 4 of 5 study sites but that one site saw a decrease in habitat by 27%. The amount, location, and longevity of new habitat appeared to be influenced by the level of human development at each site. At three of the five sites, the amount of habitat created and the time new habitat persisted were inversely related to the amount of development. Furthermore, the proportion of new habitat created in high-quality overwash was inversely related to the level of development on study areas, from 17% of all new habitat in overwash at one of the most densely developed sites to 80% of all new habitat at an undeveloped site. We also show that piping plovers exploited new habitat after the storm, with 14–57% of all nests located in newly created habitat in the 2013 breeding season. Our results quantify the importance of storms in creating and maintaining coastal habitats for beach-nesting species like piping plovers, and these results suggest a negative correlation between human development and beneficial ecological impacts of these natural disturbances.

## Introduction

Barrier islands are the product of highly dynamic environments and change naturally in response to wind, wave action, water levels, currents, and vegetation [1–4]. Coastal storms (e.g., Nor’easters, extratropical storms, tropical cyclones) often are the main agents of both short- and long-term change for these landforms [5]. These natural disturbances can transfer sand and other materials from the beach to the nearshore zone, erode backshore areas, flatten dunes, carry sediments to the back-barrier in overwash fans (or ‘washover’), and open inlets [1, 2, 6, 7]. Immediately following a major storm, a barrier island typically has a narrow, flat beach scoured of vegetation with extensive overwash [7–9]. From this post-storm geomorphic state, barrier islands with limited anthropogenic modifications have the capacity for recovery following storm events [8, 10].

Coastal storms often carry negative connotations due to loss of human lives and property (e.g., [11, 12]). Studies in the ecological literature also frequently focus on the negative consequences of storms, including wetland degradation [13], damage to coastal forests [14], and species-specific mortality and population declines [15–18]. In many areas of the world, barrier islands with important infrastructure are ‘hardened’ (i.e., stabilized with seawalls, jetties, artificial dunes, and other structural engineering techniques) or are routinely replenished with sediment from other sources with the intention of reducing negative storm impacts. In the United States, over 14% of the nation’s coastline has been hardened [19, 20]. However, these anthropogenic shoreline modifications—which are intended to protect coastlines from storm-induced erosion and flooding—can ultimately prevent some types of early successional habitats from forming [21] while more generally adversely affecting an island’s coastal ecosystems [22] and resiliency to storms [23].

Storm impacts to barrier islands can also be beneficial. For example, storm-induced overwash is important for marsh accretion, allowing these ecosystems to keep pace with changes in sea level [24]. Furthermore, species that rely on early successional habitat for one or more life history phase, such as piping plovers (*Charadius melodus*) and other shorebirds (e.g., American oystercatchers, *Haematopus palliatus*; least terns, *Sternula antillarum*) depend on storms as critical habitat creation or maintenance events [21, 25–28]. Therefore, management of coastal landforms, particularly as related to storms, may often need to consider conflicting objectives related to economic, social, and ecological issues [29].

**Fig 1:**
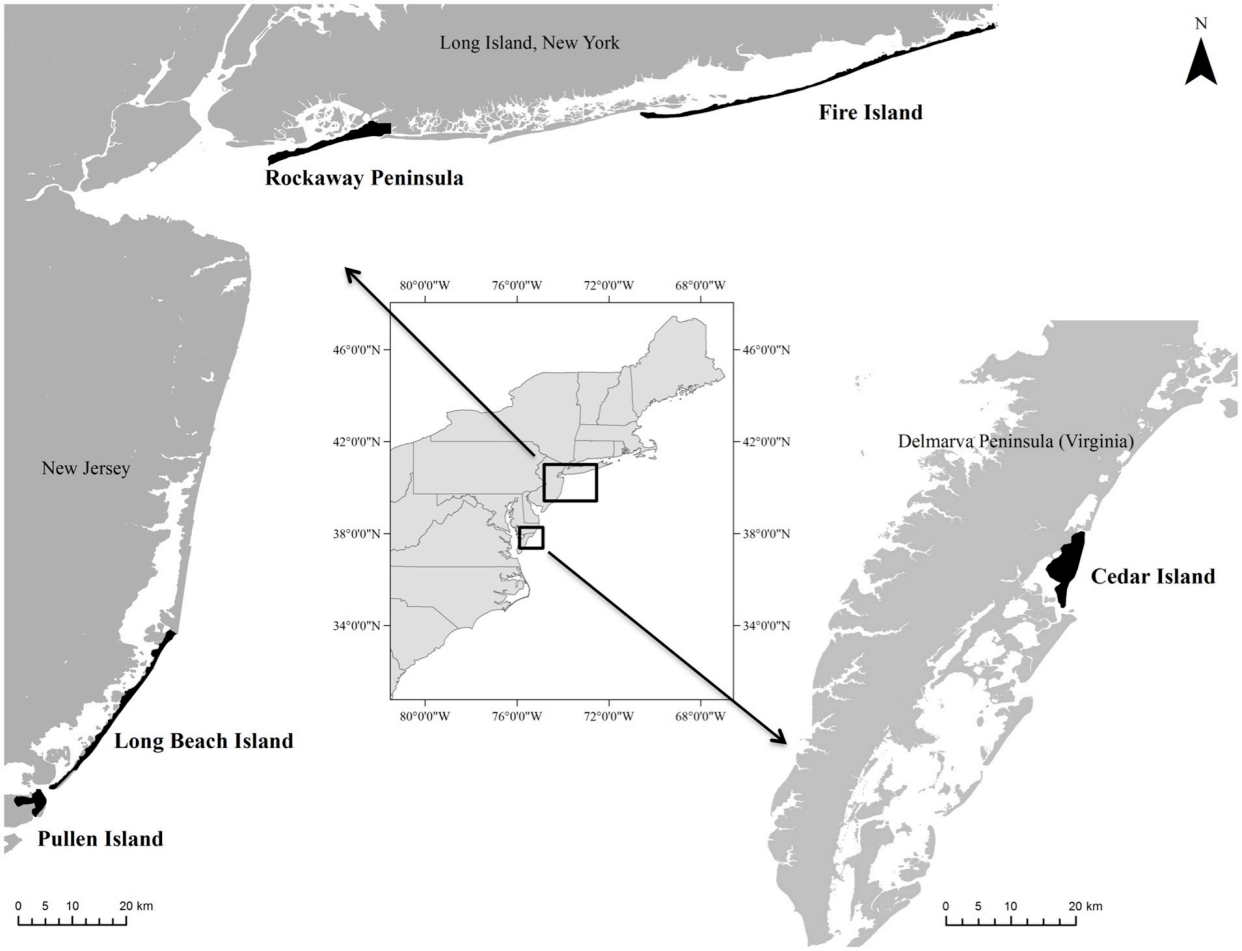
Study sites off the coasts of New York (Rockaway Peninsula, Fire Island), New Jersey (Pullen and Long Beach islands), and Virginia (Cedar Island) USA, where we modeled piping plover habitat before and after Hurricane Sandy using a Bayesian network approach.

In this study, we focus on one potential benefit of storms on coastal ecosystems, the creation and maintenance of early successional habitat used by ground-nesting shorebirds, specifically the piping plover. Our objectives were to evaluate how the quantity and location of piping plover nesting habitat changed along both anthropogenically modified and unmodified coastlines in New York, New Jersey, and Virginia (USA) during the period before and after Hurricane Sandy (Fig 1). The presence of habitat was determined for these areas in 2010 or 2011 (prior to Hurricane Sandy), immediately after the storm in November 2012, and approximately 2 years after the storm in 2014 or 2015. Exact months and years evaluated were dependent on the availability of required remotely sensed spatial datasets (e.g., lidar, orthophotographs) for each study area. We observed that habitats created immediately after the storm were quickly colonized by nesting piping plovers but that human development may have affected the amount, longevity, and location of newly created, early successional habitat. This work offers insights over a relatively broad spatial scale into the important ecological role that storms play in shaping barrier island habitats and into how human development may influence that role.

## Materials and methods

### Study species

Piping plovers are migratory shorebirds with discrete breeding populations on the Atlantic coast, the Great Lakes, and the Northern Great Plains of Canada and the United States, where they are considered either federally threatened or endangered. Here, we consider subpopulations within the Atlantic coast breeding population. These birds typically establish nests in washovers, backshore areas, and low elevation dune complexes [28, 30–34] where substrate is predominantly sandy or a mixture of sand, shell, gravel, or cobble [31, 32, 34–38] and where there is either no vegetation or sparse herbaceous vegetation [31, 32, 34, 35, 38, 39]. Breeding pairs lay up to four eggs in small depressions made in the sand, and precocial chicks hatch after approximately 27–30 days of incubation (reviewed in [40]). Adults and chicks forage largely along low-energy ocean- or bay-side intertidal zones and ephemeral pools, where they consume marine worms, arthropods, mollusks, and crustaceans [25–27]. Chicks fledge by August, when adults and fledglings begin migrating back to wintering grounds in the Caribbean and the southeastern Atlantic and Gulf of Mexico coasts (reviewed in [40, 41]).

We use piping plovers as a focal species for understanding habitat change because of their reliance on low-lying, coastal habitats and their rapid population-level responses to habitat change [28]. Furthermore, Maslo et al. [42] demonstrated that piping plovers have value as an umbrella species when considering nesting habitat vulnerability at the local scale for other species, such as American oystercatchers (*Haematopus palliatus*), black skimmers (*Rynchops niger*), and least terns (*Sterna antillarum*).

### Study sites and hurricane properties

We examined piping plover habitat and change at five sites in New York, New Jersey, and Virginia that have varying levels of shoreline development (Fig 1). The two study sites in New York include ‘Fire Island’ and the ‘Rockaway Peninsula.’ The Fire Island study site, off the coast of Long Island, USA, extends from Democrat Point (at the Fire Island Inlet) in Robert Moses State Park past the Moriches Inlet and Smith Point into Cupsogue County Park on the adjacent barrier. Fire Island itself, which comprises the entire site with the exception of Cupsogue County Park, is a micro-tidal, wave-dominated island [43]. This site is a patchwork of state, county, and federal parks interspersed with private lands and includes the Fire Island National Seashore. The approximately 50-km study area contains a mix of anthropogenically modified coastlines—with communities, housing developments, roads, and infrastructure—interspersed with federal, state, and county lands with minimal development near the primary dune-line. According to Rice [44], all ocean-front shoreline in this study area, with the exception of Democrat Point, has experienced beach replenishment (also known as beach fill or renourishment) in the last 10–15 years, and approximately 9 small areas of the coastline were armored with hard shoreline stabilization structures prior to Hurricane Sandy.

The 18-km Rockaway Peninsula study area spans from the Breezy Point Unit of the Gateway National Recreation Area and Rockaway Point to the Far Rockaways off of the Long Island coast (Fig 1). The majority of this study area is densely developed, with housing and commercial developments and infrastructure spanning all but the western-most point of the peninsula managed by the U.S. National Park Service. The shoreline along all of the Rockaway Peninsula east of Jacob Riis Park has periodically experienced beach replenishment since 1977, and approximately 14 km of the shoreline was armored with hard stabilization structures in some way prior to Hurricane Sandy [44].

The two study areas in New Jersey include ‘Long Beach Island’ and ‘Pullen Island’ (Fig 1). Pullen Island is a short (6 km), tide-dominated barrier with extensive marsh development and a narrow sandy shoreline [45]. This island forms the Little Beach Unit of the Edwin B. Forsythe National Wildlife Refuge, is undeveloped (i.e., it lacks housing communities or recreational facilities), and has experienced very little direct anthropogenic shoreline modification [44].

Long Beach Island, immediately north of Pullen Island, is a long (34 km), narrow wave-dominated island. Here, the back-barrier is characterized by fringe marshes on remnant storm-surge platforms while the ocean-side contains dune systems dissected by overwash—characteristics indicative of the strong influence waves have on shaping this island [45]. The southern-most tip of this island forms the Holgate Unit of the Edwin B. Forsythe National Wildlife Refuge. With the exception of a 1962 beach replenishment project [44], the Holgate portion of Long Beach Island has experienced little direct anthropogenic modification. The northern-most tip of Long Beach Island is also protected as part of the Barnegat Light State Park; however, this area has experienced anthropogenic modification, including a ca. 2-km terminal groin at Barnegat Inlet and four beach replenishment projects from 1962–1991 [44]. The remaining 26 km of Long Beach Island is heavily developed with residential and commercial structures. The shoreline along this portion of the study area has periodically experienced beach replenishment since the 1950s and was armored with hard shoreline stabilization structures prior to Hurricane Sandy [44].

The final study area, Cedar Island, is part of an island chain off of the Delmarva Peninsula and is bounded by the Metompkin and Wachapreague inlets (Fig 1). This site is a short (12 km), mixed-energy, tide-dominated island with a mesotidal (mean tide range ca. 2 m) coastline and an average wave height of 0.55 m ([43], reviewed in [46]). Most of Cedar Island has a wide back-barrier marsh, and extensive overwash is prevalent along much of this low elevation island (reviewed in [46]). This island has decreased in area in recent years due to high erosion rates [47]. Most of Cedar Island is jointly managed by the U.S. Fish and Wildlife Service, The Nature Conservancy, and the Commonwealth of Virginia. Although there are a few private in-holdings remaining throughout the island, it is minimally affected by development, beach replenishment, or hard shoreline stabilization structures [44].

Hurricane Sandy first made landfall near Brigantine, New Jersey on 29 October 2012 with maximum sustained winds of 130 km/hr [9, 48]. Tropical storm-force winds reached from Wallops Island, Virginia (ca. 25 km north of the Cedar Island study area) to Montauk, New York (ca. 81 km north of the Fire Island study area) at the time of landfall [9]. Maximum water levels, which include storm surge and tide, exceeded normal tide levels by 1.1–2.9 m at tide gauges from the mouth of the Chesapeake Bay to The Battery in New York [9] and were highest in New Jersey, New York, and Connecticut [49]. This storm directly impacted 24 states and was responsible for 125 deaths and over 570,000 destroyed buildings [48].

The hurricane’s effects on barrier island geomorphology were closely monitored from North Carolina to New York [9] and particularly on Fire Island [10]. These studies noted high spatial variability in storm effects, likely influenced by each area’s coastal geomorphology, offshore geology, nearshore processes, and density of human development [9]. The undeveloped barrier islands off the Delmarva Peninsula generally experienced a continued landward deposition of sand and shoreline retreat, which was the prevailing long-term trend before the storm [9]. The majority of the New Jersey coastline experienced severe dune erosion, with many areas losing 1–6 m in vertical dune-height [9]. However, positive shoreline change, beach progradation, and increases in sand volume were observed along some areas of the New Jersey shoreline, approximately 45 km north of where the storm made landfall. In this region, swash bars fused back on to the beach as part of natural beach recovery processes [9]. Overwash and dune erosion were also prevalent along the New York coastline, with an average loss in vertical dune-height from 1–2 m from the New York/New Jersey border through Fire Island [9]. On Fire Island, beaches and dunes lost more than 54% of their pre-storm volume, and dunes experienced overwash along 46% of the island [10]. Although the island experienced 7 additional storms with significant wave heights > 4 m during the winter of 2012–2013, the majority of beaches rapidly returned to pre-Sandy conditions; by April 2013, 90% of beach profiles examined had beach volumes similar to those immediately before Sandy [10]. Given the spatial variability in storm effects and ultimate geomorphological recovery from Virginia to New York, we expected to find site-specific patterns of change to beach habitats used by piping plovers in this study.

We also note that some of our inferences relating to habitat creation and availability through time were likely confounded by spatial variations in Hurricane Sandy’s direct impacts. Landfall occurred in Brigantine, New Jersey, and impacts differed across study sites due to expected changes in morphological and habitat responses [50]. Pullen and Long Beach islands were closest to the point of storm landfall at ca. 6 and 12 km, respectively, while the Rockaway Peninsula (137 km from landfall), Fire Island (192 km) and Cedar Island (234 km) were located substantially farther from the point of landfall. However, controlling for direct storm impact in this natural experiment was not possible, and the massive size of this storm produced widespread impacts associated with storm-force wind, waves, and surge [9].

#### Bayesian network

**Fig 2:**
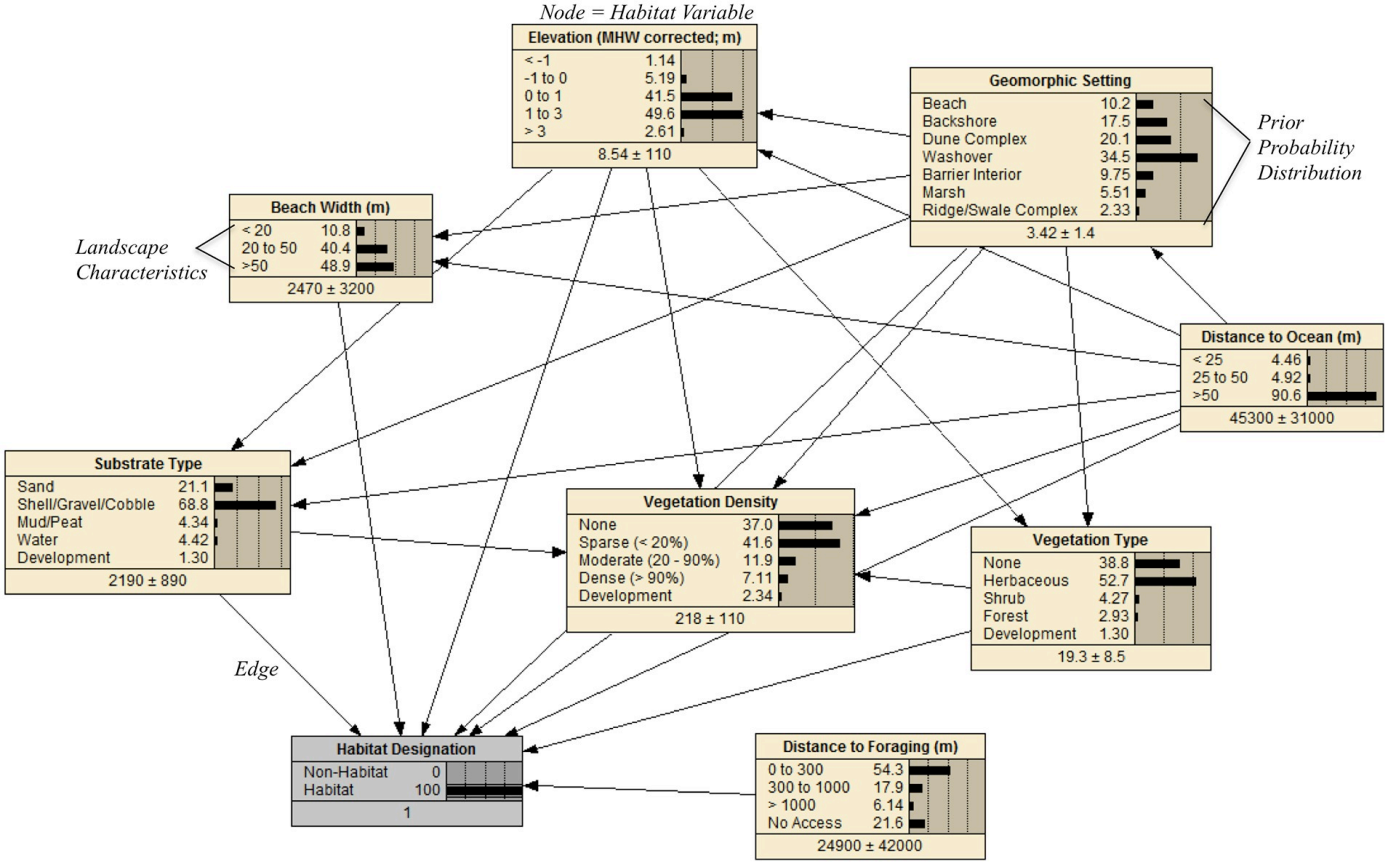
Configuration of nodes and edges in the Plover Habitat Bayesian network used to predictively map habitat for piping plovers. Prior probability distributions for this network were derived from data collected at piping plover nests and random points at sites in 2014 and 2015 using the iPlover data collection application. The network as shown in this figure illustrates prior probability distributions for instances where nests were present (i.e., characteristics associated with habitat).

We used a Bayesian network (BN) to investigate piping plover habitat availability before and after Hurricane Sandy in our study areas. An initial version of this BN and its underlying training data are described in Zeigler et al. [34]. In general, a BN is a directed acyclic graph composed of nodes and edges that organize knowledge about a system. Nodes represent variables describing relevant system components. Nodes are further broken down into discrete characteristics or, for continuous variables, discretized into bins. Edges connect nodes to convey dependencies, correlations, or causal influences among nodes (e.g., Fig 2) (e.g., Fig 2; [51]). Conditional probability distributions are calculated for each node based on empirical data according to Bayes Theorem, and the set of all possible node-value combinations forms a conditional probability table that underlies a ‘trained’ BN (e.g., Fig 2). Once trained, the BN can be used to predict the value of an unknown node given incomplete data. In such cases, the BN is used to determine the probability of observing specific states for nodes in which the true state is unknown, with epistemic uncertainty represented in the evenness of the predicted conditional probabilities [51]. BNs are generally considered powerful tools because they are robust to many common issues (e.g., missing data, multicollinearity and nonlinearity in variables) that can otherwise violate assumptions in other multivariate approaches [51, 52].

We constructed a BN for delineating piping plover habitat (‘Plover Habitat BN’; Fig 2) in Netica (version 5.12, Norsys Software Corp.) after Gieder et al.’s [53] model for Assateague Island National Seashore and Zeigler et al.’s [34] simplified regional habitat model. In our network, we connected (*i*) four nodes representing discretized continuous variables (Distance to Ocean, Beach Width, Elevation, and Distance to Foraging); (*ii*) four nodes representing categorical landcover characteristics (Geomorphic Setting, Substrate Type, Vegetation Type, Vegetation Density); and (*iii*) an output node for the probability that a specific combination of landscape characteristics in *i* and *ii* would be associated with landcover suitable for nesting (Habitat Designation; Table 1; Fig 2).

**Table 1:** Landscape variables considered as nodes in the Plover Habitat Bayesian network used to model habitat for piping plovers along the coasts of New York, New Jersey, and Virginia. Variables were associated with data collected at piping plover nest and random points through the smartphone application iPlover and were also individually mapped for our study areas. Continuous variables were discretized into bins shown in Fig 2.

*Variable*	*Variable Type*	*Possible Values*	*Definition*
1. Distance to Ocean	Continuous	0 to ∞ (meters)	When referring to points in the iPlover dataset, this is the Euclidean distance from a nest or random point to the nearest point on the mean high water (MHW) ocean shoreline. For raster coverages, this refers to the Euclidean distance from the center of each 5 x 5 m raster cell to the nearest point on the MHW ocean shoreline.
2. Beach Width	Continuous	0 to ∞ (meters)	Beach widths were measured along transects placed in 50 m intervals perpendicular to the shoreline as part of the U.S. Geological Survey’s National Assessment of Coastal Change Hazards (https://marine.usgs.gov/coastalchangehazards/). Along each transect, beach width was measured as the Euclidean distance between the MHW ocean shoreline and the nearest dune toe or anthropogenic modification (e.g., dune fencing, seawall, etc.). Points within the iPlover dataset and raster cells within spatial coverages were assigned the beach width value for the nearest transect. When no dune toe or anthropogenic modification existed along a transect, beach width for that transect was given a no data value.
3. Elevation	Continuous	- ∞ to ∞ (meters)	Denotes elevation corrected for MHW offset [55] at each nest or random point. For study area coverages, each raster cell was given the average elevation within the 5 x 5 m area covered by that cell.
4. Distance to Foraging	Continuous	0 to ∞ (meters)	When referring to points in the iPlover dataset, this is the least cost path distance from a point to the nearest foraging area (e.g., intertidal zone along inlets or sandy bay-side beaches, ephemeral pools, etc.). Foraging areas did not include ocean intertidal zones. For raster coverages, distance to foraging refers to the least cost path distance from the center of each 5 x 5 m raster cell to the nearest foraging area. The least cost path routine assumed that areas of moderate or dense vegetation, water, or development served as barriers that blocked access to foraging grounds. Distances, therefore, assume paths that wind around these barriers. When no possible route existed between a nest, random point, or raster cell to a foraging area, this variable was given a fill value denoting ‘no access’ to foraging grounds.
5. Geomorphic Setting	Categorical	Beach; Backshore; Dune Complex; Washover; Barrier Interior; Marsh; Ridge/Swale	The geomorphic setting that best described the location of a nest, random point, or 5 x 5 m raster cell. More detailed definitions of the possible categorical values are given in Zeigler et al. [34].
6. Substrate Type	Categorical	Sand; Shell/Gravel/Cobble; Mud/Peat; Water; Development	The substrate type that best described the 5 x 5 m area surrounding a nest or random point in the iPlover dataset or a raster cell in spatial coverages. We selected Development as the substrate type for any point or cell that fell within areas obviously influenced by anthropogenic activities (e.g., housing developments, paved roads or parking lots, recreational sports fields, etc.). More detailed definitions of the possible categorical values are given in Zeigler et al. [34].
7. Vegetation Type	Categorical	None; Herbaceous; Shrub; Forest; Development	The vegetation type that best described the 5 x 5 m area surrounding a nest or random point in the iPlover dataset or a raster cell in spatial coverages. We selected Development as the vegetation type for any point or cell that fell within areas obviously influenced by anthropogenic activities (e.g., housing developments, paved roads or parking lots, recreational sports fields, etc.). More detailed definitions of the possible categorical values are given in Zeigler et al. [34].
8. Vegetation Density	Categorical	None; Sparse (0–20% coverage); Moderate (20–90%); Dense (> 90%); Development	The approximate percentage of the 5 x 5 m area surrounding a nest or random point in the iPlover dataset or a raster cell that was covered by vegetation. We selected Development as the vegetation density for any point or cell that fell within areas obviously influenced by anthropogenic activities (e.g., housing developments, paved roads or parking lots, recreational sports fields, etc.). More detailed definitions of the possible categorical values are given in Zeigler et al. [34].

We followed guidelines for the development of BN structure outlined in Marcot et al. [52]. Network structure, including nodes and their connections, was based on an extensive review of the published literature [28, 30–39] and consultation with species and geomorphology experts. In particular, structuring node correlations through expert consultation is recommended over using case data to automatically establish correlations in order to avoid data overfitting and spurious statistical correlations (reviewed in [52]). We also ensured to the extent possible that no child node had more than three parent nodes (with the exception of the Vegetation Density and Habitat nodes), all nodes were observable or quantifiable, and the number of discrete states was minimized to five or fewer (with the exception of Geomorphic Setting) [52]. Prior probability distributions and the conditional probability table for each node in the BN were derived from field data supplemented with remotely sensed information (see sub-section *Training Data*). Hence, while the BN approach does not specifically tease apart ecological and biological process—such as explicitly examining what led to individual bird nesting decisions—it frames hypotheses tests for the importance of factors at a macroscopic scale that have been proposed as being causal to these decisions.

We examined the accuracy or classification power of the BN using two different approaches and evaluated the BN’s sensitivity to individual nodes. For BN validation, we first determined the receiver operating characteristic (ROC) curve, which calculates the prediction accuracy of the network over the full continuum of prediction thresholds (instead of over an arbitrary probability threshold like 0.5) [52]. We also calculated the BN’s predictive accuracy through 10-fold cross-validation in the Python module CVNetica according to Fienen and Plant [54]. For the node sensitivity analysis, we systematically removed nodes for each variable contained in the model and recalculated the mean error rate through 10-fold cross-validation. A full description of the methodologies used to assess the BN’s predictive accuracy and the results of this testing are contained in Supporting Information (S1 File). In brief, validation demonstrated that the BN had high accuracy in predictions of landcover characteristics associated with piping plover nest habitat selection (mean error rate = 0.23; ROC curve in top-left quadrant of plot with AUC = 0.90; S1 Fig). The network was not sensitive to the removal of any one node, with the removal of the node for Beach Width resulting in the largest change in mean error rate (error rate = 0.18; S1 Table).

#### Training data

We developed the smartphone application ‘iPlover’ [56] and partnered with individuals representing the U.S. Fish and Wildlife Service, U.S. National Park Service, several state wildlife or environmental agencies, and several private conservation organizations [34] to collect data on piping plover nesting habitat-use patterns that could be used to parameterize the Plover Habitat BN. Under our collection protocol, each iPlover user, after finding a nest in the course of monitoring efforts, used the smartphone’s internal sensors within iPlover to record the nest’s geolocation coordinates and observation date and time. The user completed a simple habitat assessment by assigning categorical values to a fixed set of variables listed in iPlover. Habitat variables considered included Geomorphic Setting, Substrate Type, Vegetation Type, and Vegetation Density (Table 1). iPlover records were locally stored on smartphones while users were in the field and uploaded to a centralized database as soon as internet connectivity was available. An identical protocol was employed at the locations of random, non-nesting points disseminated to our partners at the start of each breeding season. For additional details on the smartphone application, protocol, and dataset, see Thieler et al. [56], Zeigler et al. [34], and Sturdivant et al. [57].

The iPlover dataset used for model training in this study contained habitat assessments at 287 nest and 269 random points collected during the breeding seasons (March to July) of 2014 and 2015 at Fire Island (71 nest, 40 random), the Rockaway Peninsula (40 nest, 39 random), Pullen Island (43 nest, 54 random), Long Beach Island (50 nest, 34 random), Cedar Island (49 nest, 59 random), Cobb Island (1 nest, 10 random; Virginia, USA), and Smith Island (33 nest, 33 random; Virginia, USA). All data are available at: http://dx.doi.org/10.5066/F70V89X3. Within this dataset, we assumed that landcover characteristics of nest points were associated with habitat and characteristics of random points were associated with unsuitable landcover, or non-habitat. Because we used nest presence as a proxy for habitat instead of, for example, egg fate or fledging rate, we cannot make conclusions regarding whether selected habitat was optimal for nesting or acted as an ecological trap [58].

In order to derive prior probability distributions for the remaining continuous variables in the BN, we supplemented the dataset with additional landscape data. We refer to this dataset as the ‘supplemented iPlover dataset’. To do this, we used remotely sensed lidar and aerial photographs captured during the appropriate study periods (S2 Table) to create raster layers covering an entire study area for each of the habitat variables considered in the Plover Habitat BN [59]. All spatial data are available in Sturdivant et al. [60]. In ArcGIS, we overlaid the iPlover dataset points over these raster layers and used the ‘Extract Multi-Values to Points’ tool in ArcToolBox. This tool extracts the cell values from the underlying raster layers at locations specified in a point feature class (i.e., the iPlover points) and records those values to the attribute table of the point feature class.

### Spatial analyses

The trained network was then used to map habitat according to Zeigler et al. [34]. For each study area and year, we used orthoimagery and lidar (S2 Table) to create eight geographic information system (GIS) layers in ArcGIS (version 10.4) that represented each input node in the Plover Habitat BN. We combined the eight layers to form a single GIS layer, where every 5x5 m cell had an attribute for each of the input nodes for a given year. Spatial data and a detailed description of how each individual GIS layer was created are provided in Zeigler et al. [59], and GIS layers are available in Sturdivant et al. [60]. For this study, we ultimately derived 120 individual GIS layers (8 GIS layers x 3 years x 5 study sites) and 15 combined GIS layers for the study sites and years considered (5 study sites x 3 years). The attribute table associated with each combined GIS layer in ArcGIS showed every unique combination of the eight landscape variables. This table was used as a case file, where every row in the table presented an individual case for probabilistic interpretation by the BN. We ran the case file through the trained Plover Habitat BN with the Process Cases function in Netica. This analysis generated a probability value for the Habitat Designation node for each combination of characteristics that we joined back to the original attribute file in ArcGIS for mapping purposes.

The final result was a habitat map for each year and study site, where every 5 x5 m raster cell had a probability reflecting that cell’s likelihood of containing piping plover habitat given its underlying landscape characteristics in a given year. In post-processing, we defined thresholds for which probabilities indicated the presence of habitat based on the Intergovernmental Panel on Climate Change’s (IPCC’s) likelihood scale [61]. Under this scale, a landscape cell was considered ‘very likely habitat’ if it had a probability ≥ 0.90 of being habitat, ‘likely habitat’ with a probability 0.66–0.90, ‘uncertain’ with a probability 0.33–0.66, and ‘unlikely habitat’ with a probability ≤ 0.33. Landscape cells with probabilities ≥ 0.66 were considered habitat, while cells with probabilities ≤ 0.33 were considered non-habitat. We show that these thresholds proved meaningful for identifying the likelihood of actual piping plover nesting in Supporting Information (S1 File; S3 Table).

Landcover in the probability range spanning 0.33–0.66 could have two interpretations. In the first interpretation, the combination of habitat variables was either not present in the supplemented iPlover dataset or there was insufficient information in the ‘case’ presented to the BN for analysis. In these instances, there was not enough information to make an accurate prediction, and the resulting model prediction—which is truly uncertain—was centered tightly around 0.50. In the second interpretation, as probabilities move from 0.50 toward 0.66 or 0.33, the combination of landcover characteristics may be associated with landcover of marginal suitability. Where possible, we differentiate between what are uncertain model predictions based on a lack of information (*p* = 0.50) and where landcover may be of marginal suitability (0.50 < *p* > 0.66; 0.33 < *p* > 0.50).

For each study site, we then compared the pre-Sandy to the post-Sandy habitat maps and the post-Sandy to the ca. 2-year post-Sandy habitat maps to denote areas of habitat creation and loss. For example, when landcover in a given 5x5 m cell transformed from habitat to non-habitat or from habitat to uncertain between the pre-Sandy and the post-Sandy periods, we classified that change as a loss in habitat that occurred during that period. If landcover transformed from non-habitat to habitat or from uncertain to habitat during that period, we classified that change as habitat creation. We evaluated habitat change between the post-Sandy and the ca. 2-year post-Sandy periods in the same manner. In general, we assume that substantial changes in habitat availability that occurred between study periods (2010/2011 to 2012, 2012 to 2014/2015) were the result of Hurricane Sandy and each barrier island’s subsequent recovery from that event. Because the Bayesian network only evaluates the presence of habitat and not the driver of habitat creation, events other than Hurricane Sandy (e.g., management or habitat restoration, nor’easters, other tropical storms) may have also contributed to our observed changes in habitat amount and location. However, habitat changes not driven by Hurricane Sandy were likely minimal. According to storm records by the National Oceanic and Atmospheric Administration [62], the mid-Atlantic and northeastern United States experienced the effects of tropical storm Irene (2011) prior to Hurricane Sandy as well as extratropical storm Andrea (2013) after Hurricane Sandy. Records on winter storms (or nor’easters) are less organized, but databases by the National Weather Service (available https://w2.weather.gov/climate/) suggest that one nor’easter in 2010 and two nor’easters in 2012 could have affected habitat prior to Hurricane Sandy while one nor’easter two weeks after Hurricane Sandy (2012) and two nor’easters in 2014 could have affected habitat. Other known restoration/management activities are discussed in the Results and Discussion.

Because development can influence the geomorphological behavior and storm response of barrier islands ([19, 63, 64], reviewed in [65, 66]), we placed our habitat modeling results in the context of the degree of development in study areas. To do this, we hand-digitized a rough outline or ‘footprint’ in ArcGIS that encompassed areas of development based on the orthoimagery [59, 60] for each study area. Areas within the development footprint included beach habitats directly abutting housing developments and paved recreational infrastructure. This development footprint covered 88% of 33-km^2^ Long Beach Island, 73% of 25-km^2^ Rockaway Peninsula, and 31% of 32-km^2^ Fire Island. Development was not present on Cedar or Pullen islands as of 2014. Therefore, our study sites represented a spectrum from densely developed (Long Beach Island, Rockaway) to moderately developed (Fire Island) to undeveloped (Cedar and Pullen islands). We report area and change in habitat both inside and outside of these development footprints, with the caveat that habitat within footprints may be functionally unsuitable as a result of human disturbance (e.g., due to human-plover interactions).

Finally, we also examined where habitat was created between the pre- and post-Sandy periods within different geomorphic settings in each study area. To do this, we used the Geomorphic Setting raster layer [59, 60] for the post-Sandy period for each study area and calculated the area of habitat gained within that setting between the pre- and post-Sandy periods.

For reference, we discuss changes in annual piping plover population size, average productivity, and locations of piping plover nests at each study site. Nest locations in 2014 and 2015 were contained in the iPlover dataset [34]. All population abundance and productivity data and nest locations (when available) prior to 2014/2015 were obtained from the unrelated research and monitoring efforts of outside partners [67–69]. Piping plover nests presented or discussed in this study do not necessarily represent all nests found in a given study area for that year. For example, portions of the Rockaway Peninsula were not surveyed by our partners, and nests located in those unsurveyed areas are not accounted for here. Nest locations contained here are those that were available to us and our partners at the time of this study. Methodology for nest and population surveys is summarized elsewhere [67–70], and nest surveys were primarily conducted in the undeveloped portions of study areas considered here.

## Results and discussion

### Habitat change following Hurricane Sandy

The amount of piping plover nesting habitat increased immediately following Hurricane Sandy at four of five sites, adding 0.1 km^2^–1.6 km^2^ (or a 9–300% increase) of new habitat depending on the site (Tables 2 and 3). Previous studies have also illustrated the importance of storms for the creation and maintenance of early successional coastal habitats on Long Island, New York [28, 71], the North Brigantine Natural Area, New Jersey [72], and on Assateague Island, Maryland [73]. However, these studies used increases in piping plover abundance or productivity to approximate for changes in breeding habitat (but see [28] for habitat estimates on Long Island). Our study uniquely quantifies the increase in habitat following a storm across entire islands and considers a broad spatial scale. Patterns of habitat creation are further supported by Rice [20], who found that Hurricane Sandy likely increased piping plover habitat along 103 miles of sandy beaches, at 33 new tidal inlet locations, and in new bare sand habitat at 4 closed inlets.

**Table 2:** Predicted habitat, according to the Plover Habitat Bayesian network^1^, for piping plover nesting before Hurricane Sandy, immediately following Sandy, and ca. 2 years post-Sandy at study areas in New York, New Jersey, and Virginia, USA. For reference, we indicate the number of nests^2^ that were established in what the model predicted to be habitat, non-habitat, or uncertain landcover (where a suitability designation could not be made). Sites are listed in decreasing level of anthropogenic development.

Imagery Acquisition Year (nest data breeding season)	Predicted Habitat Area–All Habitat (km^2^)	Predicted Habitat Area–Habitat in patches > 0.1 km^2^ (km^2^)	Number of Nests (% of nests) in:		
			*Habitat*	*Landcover where Suitability Uncertain* *[Landcover where p = 0*.*5]*^3^	*Non-Habitat*
*Long Beach Island*, *New Jersey*					
Pre-Sandy: Spring 2010 (breeding season 2010)	0.2	0.2	0	17 (89%) [14 had *p* = 0.5]	2 (11%)
Post-Sandy: Oct. 2012 (breeding season 2013)	0.7	0.4	8 (57%)	6 (43%) [5 had *p* = 0.5]	0
ca. 2-years Post-Sandy: April 2014 (breeding season 2014)	1.5	1.5	14 (100%)	0	0
*Rockaway Peninsula*, *New York*					
Pre-Sandy: Spring 2011 (breeding season 2011)	1.1	0.9	11 (79%)	3 (21%) [2 had *p* = 0.5]	0
Post-Sandy: Oct. 2012 (breeding season 2013)	1.2	1.0	15 (71%)	6 (29%) [0 had *p* = 0.5]	0
ca. 2-years Post-Sandy: April 2014 (breeding season 2014)	1.0	0.9	11 (65%)	4 (23%) [3 had *p* = 0.5]	2 (12%)
*Fire Island*, *New York*					
Pre-Sandy: Oct. 2011 (breeding season 2012)	1.4	1.0	-----------	-----------	-----------
Post-Sandy: Oct. 2012 (breeding season 2013)	3.0	2.6	-----------	-----------	-----------
ca. 2-years Post-Sandy: April 2015 (breeding season 2015)	2.5	2.1	37 (79%)	5 (10.5%) [3 had *p* = 0.5]	5 (10.5%)
*Pullen Island*, *New Jersey*					
Pre-Sandy: Spring 2010 (breeding season 2010)	0.1	0.1	2 (11%)	16 (89%) [15 had *p* = 0.5]	0
Post-Sandy: Oct. 2012 (breeding season 2013)	0.4	0.3	12 (36%)	21 (64%) [14 had *p* = 0.5]	0
ca. 2-years Post-Sandy: April 2014 (breeding season 2014)	0.6	0.6	22 (96%)	1 (4%) [1 had *p* = 0.5]	0
*Cedar Island*, *Virginia*					
Pre-Sandy: Spring 2011 (breeding season 2011)	2.2	2.2	-----------	-----------	-----------
Post-Sandy: Spring 2013 (breeding season 2013)	1.6	1.5	42 (74%)	15 (26%) [14 had *p* = 0.5]	0
ca. 2-years Post-Sandy: April 2014 (breeding season 2014)	1.4	1.4	28 (100%)	0	0

^1^Habitat was defined as landcover that had a ≥ 0.66 probability of being habitat for piping plover nesting, according to the Plover Habitat Bayesian network. Non-habitat was defined as landcover that had a ≥ 0.33 probability of being habitat for piping plover nesting. Landcover considered as likely as not habitat was defined as landcover that had a 0.33–0.66 probability of being habitat for piping plover nesting.

^2^Sources for nest data: (1) Rockaway and Fire Island: K. Jennings, 2010–2016. Long Island colonial waterbird and piping plover survey results, annual reports for 2010 to 2016. New York State Department of Environmental Conservation; (2) Long Beach and Pullen islands: Pover, T. and C. Davis. 2010–2016. Piping plover nesting results in New Jersey, annual reports for 2010 to 2016. Conserve Wildlife Foundation of New Jersey and New Jersey Division of Fish and Wildlife; (3) Cedar Island: Boettcher, R. 2010–2016. Piping plover nesting results in Virginia, annual reports for 2010 to 2016. Virginia Department of Game and Inland Fisheries. We were unable to obtain georeferenced nest data for Fire Island in the 2012 and 2013 breeding seasons and for Cedar Island in the 2011 breeding season.

^3^For nests located in areas where suitability is uncertain, a habitat probability of 0.5 suggests that the model did not have training data for that particular combination of habitat variables or that there was missing information in the underlying combination of landcover variables associated with the nest point. Therefore, the best interpretation would be that the model was unable to make a prediction based on a lack of data, not that nests were found in marginal (lower suitability) habitat.

**Table 3:** Changes in piping plover habitat availability^1^ for landcover conditions present before Hurricane Sandy, immediately following Sandy, and ca. 2 years post-Sandy at study areas in New York, New Jersey, and Virginia USA. For reference, we indicate the number of nests that were established in habitat where landcover became habitat, became non-habitat, did not change in suitability, or became uncertain in suitability level between the pre-Sandy to post-Sandy (nests: 2013 breeding season) and between the post-Sandy and ca. 2-year post-Sandy (nests: 2014/2015 breeding season) study periods. Sites are listed in decreasing level of human development.

Period of Change	Area of Landcover that Became Habitat (km^2^)	Area of Landcover that Became Non-Habitat (km^2^)	Area of Landcover where Suitability became Uncertain (km^2^)	Net Change in Amount of Habitat (km^2^) [% change]	Number of Nests (% of nests) in landcover that:			
					*Became Habitat*	*Became Non-Habitat*	*Did Not Change in Suitability*	*Became Uncertain in Suitability Level*
*Long Beach Island*, *New Jersey*								
Pre-Sandy to Post-Sandy	0.6	0.0	0.1	+0.5 [+250%]	8 (57%)	0	5 (36%)	1 (7%)
Post-Sandy to ca. 2-years Post-Sandy	1.0	0.0	0.2	+0.8 [+114%]	8 (57%)	0	6 (43%)	0
*Rockaway Peninsula*, *New York*								
Pre-Sandy to Post-Sandy	0.6	0.1	0.4	+0.1 [+9%]	11 (52%)	0	10 (48%)	0
Post-Sandy to ca. 2-years Post-Sandy	0.3	0.1	0.4	-0.2 [-17%]	4 (23.4%)	2 (11.8%)	9 (53%)	2 (11.8%)
*Fire Island*, *New York*								
Pre-Sandy to Post-Sandy	2.1	0.1	0.5	+1.6 [+114%]	-----------^2^	-----------	-----------	-----------
Post-Sandy to ca. 2-years Post-Sandy	1.1^3^	0.2	1.2	-0.5 [17%]	17 (36%)	0	28 (60%)	2 (4%)
*Pullen Island*, *New Jersey*								
Pre-Sandy to Post-Sandy	0.4	0.0	0.1	+0.3 [+300%]	12 (36%)	0	19 (58%)	2 (6%)
Post-Sandy to ca. 2-years Post-Sandy	0.5	0.1	0.1	+0.2 [+50%]	15 (65%)	0	8 (35%)	0
*Cedar Island*, *Virginia*								
Pre-Sandy to Post-Sandy	0.4	0.0	1.0	-0.8 [-27%]	8 (14%)	0	38 (67%)	11 (19%)
Post-Sandy to ca. 2-years Post-Sandy	0.4	0.0	0.6	-0.2 [-13%]	7 (25%)	0	21 (75%)	0

^1^We designated changes in suitability according to the following criteria: (1) ‘Became Habitat’, landcover in a given cell transformed from As Likely As Not Habitat to Habitat or from Non-Habitat to Habitat; (2) ‘Became Non-Habitat’; landcover in a given cell transformed from Habitat to Non-Habitat; (3) ‘Did Not Change in Suitability’, the suitability level of landcover in a given cell (whether Non-Habitat, Habitat, or As Likely As Not Habitat) did not change or ‘Non-Habitat’ became ‘As Likely As Not Habitat’ (or vice versa), and (4) ‘Became Uncertain in Suitability Level’, the suitability level of landcover in a given cell transformed from Habitat to As Likely As Not Habitat. For example, if landcover in a landscape cell that was Non-Habitat in 2010 was considered Habitat in 2012 after Hurricane Sandy, we designated this as a location that Became Habitat. Because we were primarily interested in the creation and loss of habitat, we designated a change from As Likely As Not Suitable to Non-Habitat and vice versa as ‘No Change in Suitability’.

^2^Dashed lines indicate that data necessary to complete this analysis were unavailable.

^3^The creation of a dredge material area and a conservation restoration zone resulted in 0.1 km^2^ and 0.2 km^2^, respectively, of new habitat between 2012 and 2015 for the Fire Island study area.

**Fig 3:**
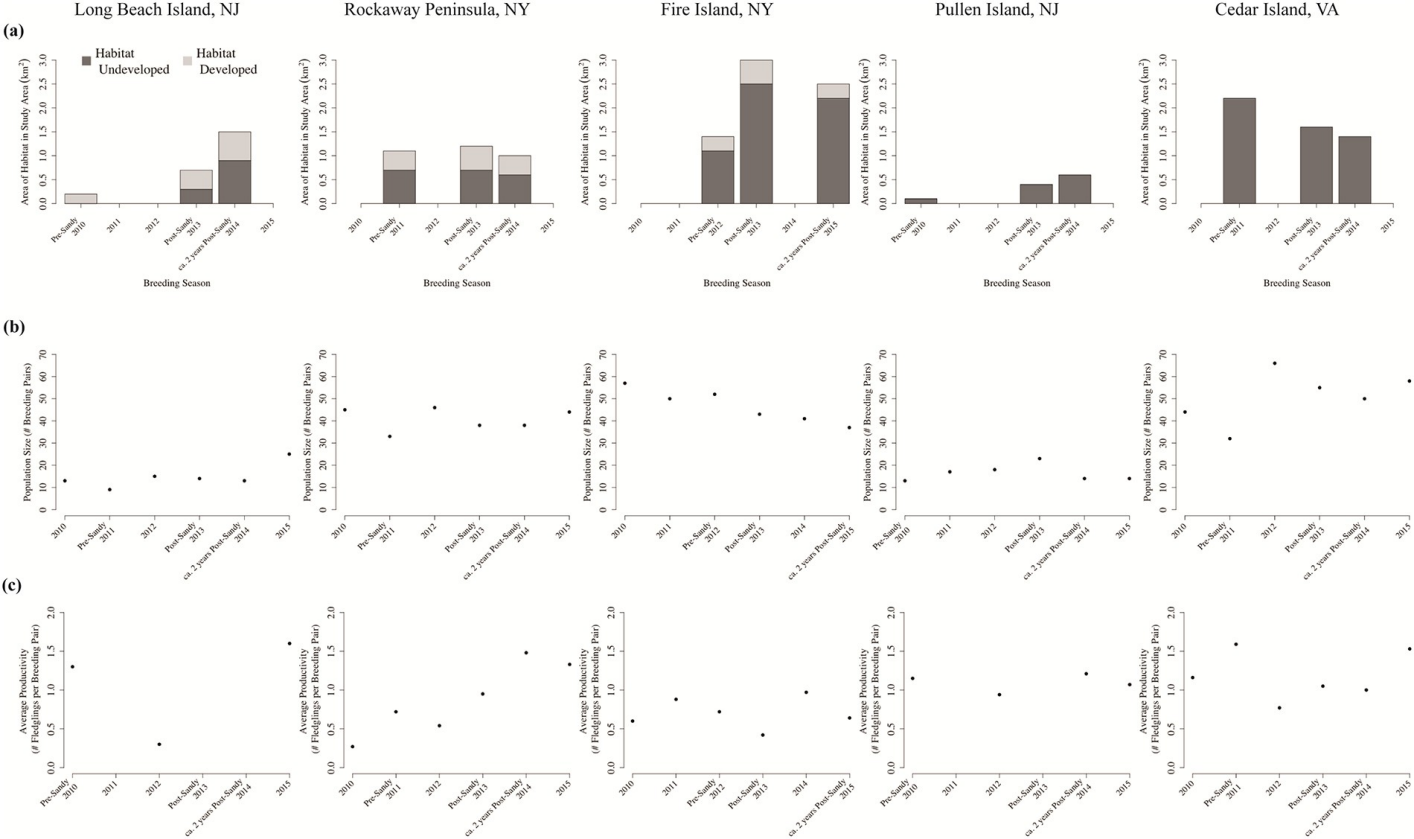
(a) Changes in the area of piping plover habitat, as predicted by the Plover Habitat Bayesian network, before Hurricane Sandy, immediately after the storm, and ca. 2 years after the storm at study sites in New York (Rockaway Peninsula, Fire Island), New Jersey (Long Beach and Pullen islands), and Virginia (Cedar Island). Sites are shown in order of proportion of human development on the study site, from Long Beach Island (most developed) to Cedar Island (undeveloped), and bars are divided as the area of habitat found along developed portions of the coastline (i.e., where housing communities, recreational infrastructure, or other human structures directly abut the shoreline; light portions of bars) and habitat found along undeveloped portions of the coastline (dark portions of bars). We also show piping plover (b) population size and (c) average productivity at each study site to illustrate how population dynamics changed with habitat amount after the storm. Population data were collected by outside partners as part of unrelated research and monitoring efforts [67–69].

**Fig 4:**
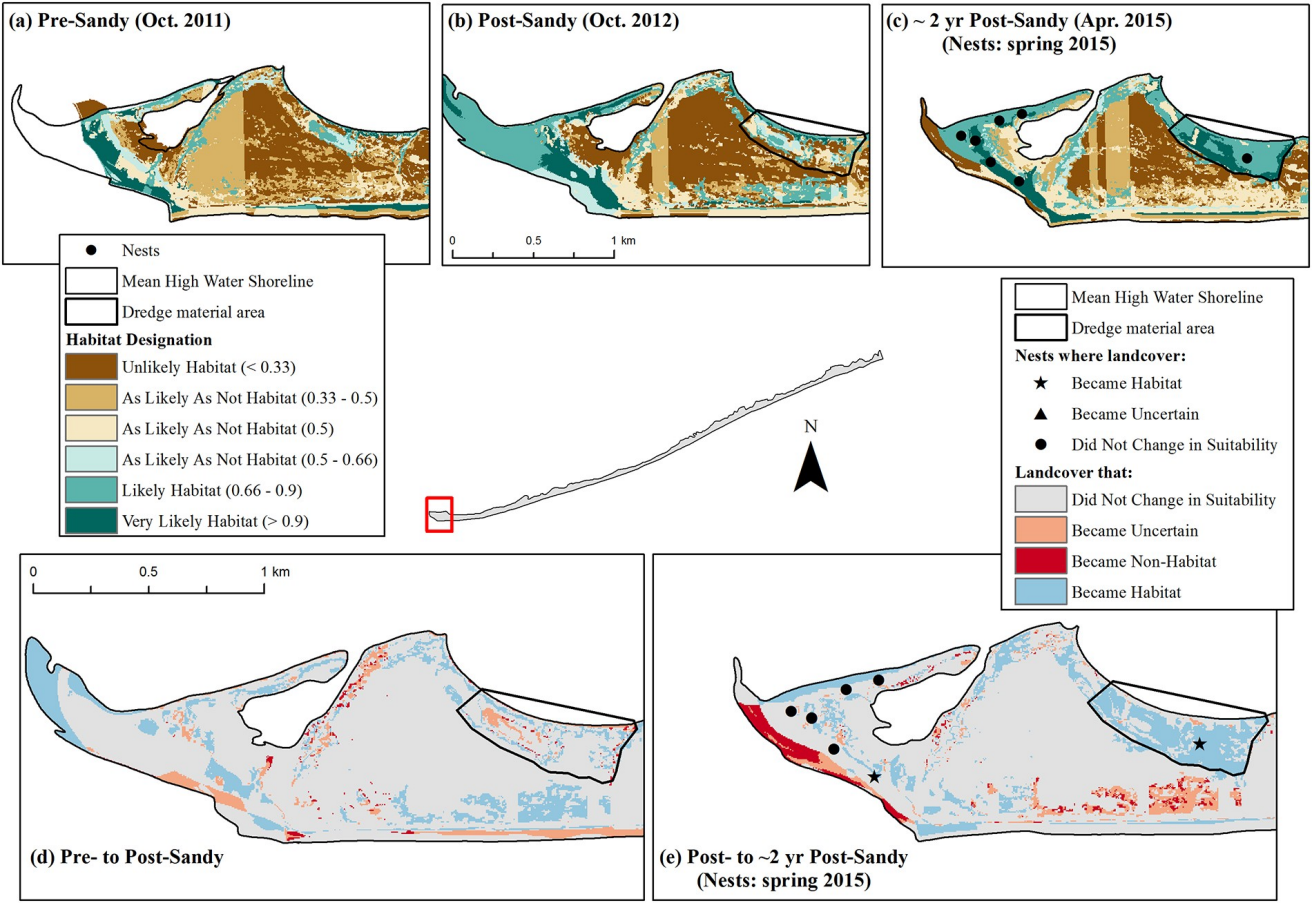
Examples of habitat change before Hurricane Sandy, immediately following Sandy, and ca. 2 years post-Sandy on the Fire Island, New York, study area^1^. Maps (a)-(c) show habitat in the year corresponding with the aerial imagery used for analysis, with piping plover nest points corresponding to the breeding season for which habitat was available^2^. Map (d) shows changes in habitat that ostensibly resulted from Hurricane Sandy, while map (e) shows changes in habitat since the hurricane occurred^3^. ^1^In these maps, the habitat designation was predicted according to the Plover Habitat Bayesian network. Landcover that was very likely habitat had a ≥ 0.90 probability of being habitat, landcover likely habitat had a probability of 0.66–0.90, landcover as likely as not habitat had a probability of 0.3–0.66, and landcover unlikely habitat had a probability ≤ 0.33. We further differentiated the ‘as likely as not habitat’ category to show (1) areas of true model uncertainty (*p* = 0.5), where the combination of habitat variables in a given landscape pixel was either not present in the supplemented iPlover dataset or where there was missing information in the ‘case’ presented to the BN for analysis in that pixel and (2) areas where landcover may be of marginal suitability (0.50 < *p* < 0.66; 0.33 < *p* < 0.50). ^2^Nest locations contributed by [68]. ^3^In these maps, habitat change was predicted by the Plover Habitat Bayesian network, where (d) considers change from the pre- to the post-Sandy study periods and (e) considers change from the post- to the 2 years post-Sandy study periods. Landcover that ‘became habitat’ transformed from as likely as not habitat or non-habitat to habitat; landcover that ‘became uncertain’ transformed from habitat to as likely as not habitat; and landcover that ‘became non-habitat’ transformed from habitat to non-habitat. Landcover that ‘did not change in suitability’ was either habitat, as likely as not habitat, or non-habitat in both study periods considered OR transformed from as likely as not habitat to non-habitat (or vice versa) between study periods.

We observed the greatest total increase in habitat at Fire Island; the amount of habitat increased from 1.4 km^2^ pre-Sandy to 3.0 km^2^ immediately post-Sandy (Fig 3a). Areas where habitat was created can be seen in the example shown in Fig 4. Here, the amount of landcover predicted to be habitat (*p* ≥ 0.66; shown in shades of medium and dark green) increased from panel (a) to (b), and swaths of blue in panel (d) show where new habitat was created immediately after the storm (Fig 4). Approximately 2 years after the storm, the amount of habitat at Fire Island was 2.5 km^2^ (Tables 2 and 3). This marked a decline from post-Sandy habitat levels, but habitat amount ca. 2 years post-Sandy remained higher than pre-Sandy levels (Fig 3a). Examples of areas where habitat was lost between the post- and ca. 2 years post-Sandy periods are shown in pink and red in Fig 4e.

U.S. Army Corps of Engineers (USACE) and landowner actions between the post- and ca. 2 years post-Sandy periods affected habitat change during that period on Fire Island. Mechanical closures of two breaches and placement of multiple rows of sand fence along undeveloped beaches [20] likely inhibited the amount of post-storm habitat growth (such as occurred on undeveloped portions of Long Beach Island). However, the model also predicted 0.1 km^2^ of piping plover nesting habitat at Democrat Point in an area where dredge material was stockpiled and graded adjacent to a steep intertidal zone (location shown in Fig 4). In addition, the USACE mechanically created two experimental habitat enhancement zones within Smith County Park prior to the 2015 nesting season in an effort to partially offset habitat loss due to beach nourishment and artificial dune construction (S. Papa, U.S. Fish and Wildlife Service, pers. comm.). Our model predicted that these activities created an additional 0.2 km^2^ of nesting habitat (based on orthoimagery captured in April 2015). However, one of these zones revegetated before the conclusion of the 2015 breeding season, and contouring along the other restoration zone—intended to create moist foraging areas—quickly disappeared (S. Papa, U.S. Fish and Wildlife Service, pers. comm.). As a result, far fewer nesting pairs have colonized this area than expected [74]. Thus, human activities may have impeded natural growth of some habitats while also creating habitat that may have degraded shortly after imagery used in this analysis was taken. A larger decrease in habitat—an additional loss of 0.3 km^2^ on top of the 0.5 km^2^ lost as documented in this modeling work—could have been evident between the post- and ca. 2 years post-Sandy periods at Fire Island without these activities.

**Fig 5:**
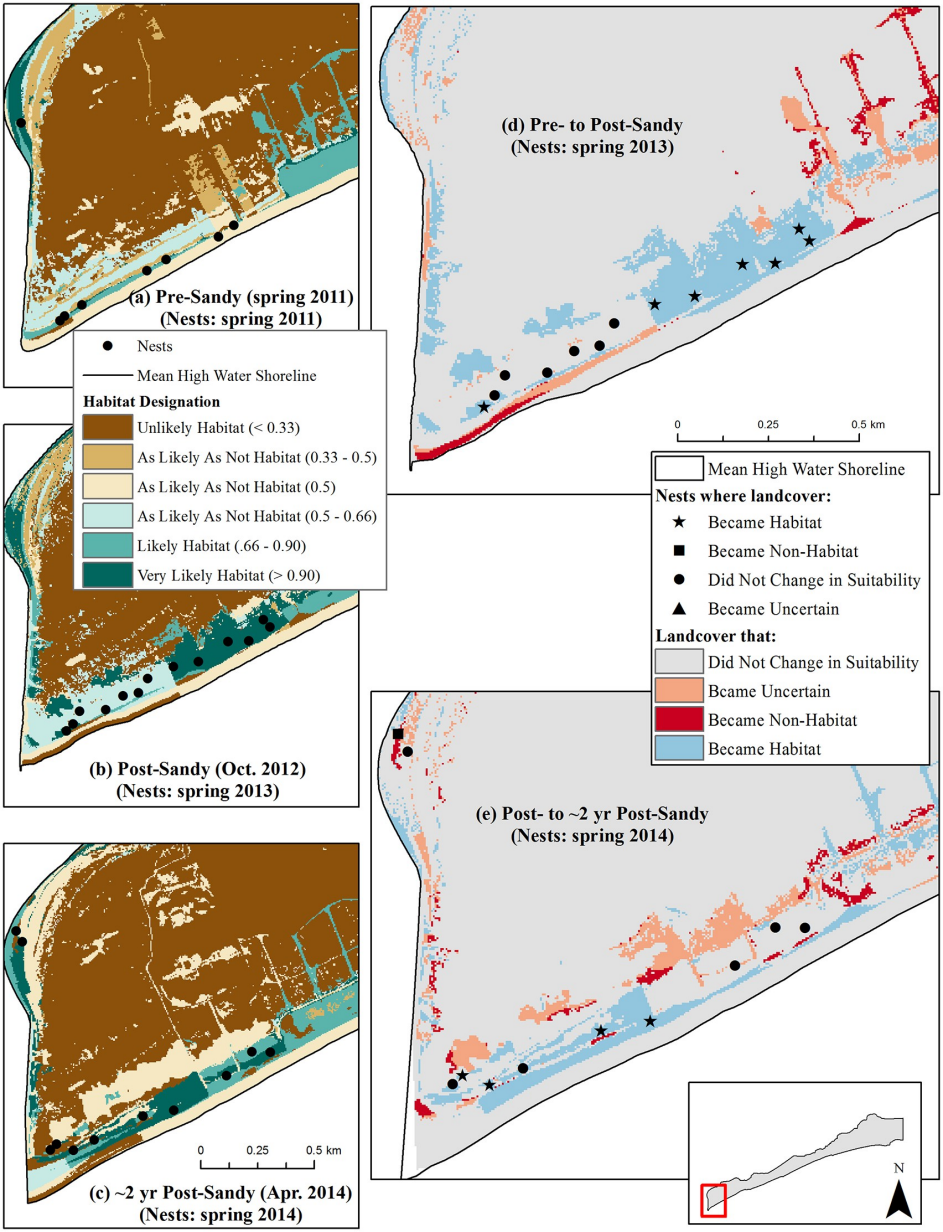
Examples of habitat change before Hurricane Sandy, immediately following Sandy, and ca. 2 years post-Sandy within the Rockaway Peninsula, New York, study area. Maps (a)-(c) show habitat in the year corresponding with the aerial imagery used for analysis, with piping plover nest points corresponding to the breeding season for which habitat was available^1^. Map (d) shows changes in habitat that ostensibly resulted from Hurricane Sandy, while map (e) shows changes in habitat since the hurricane occurred^2^. ^1^Nest points contributed by [68] ^2^See footnotes accompanying Fig 4.

We observed a similar general pattern to that of Fire Island on the Rockaway Peninsula. Habitat increased immediately following Hurricane Sandy to 1.2 km^2^ but then declined below pre-Sandy levels to 1.0 km^2^ by the ca. 2 years post-Sandy period (Tables 2 and 3; Fig 3a). Examples of where habitat was created and lost for this study area are shown in Fig 5.

**Fig 6:**
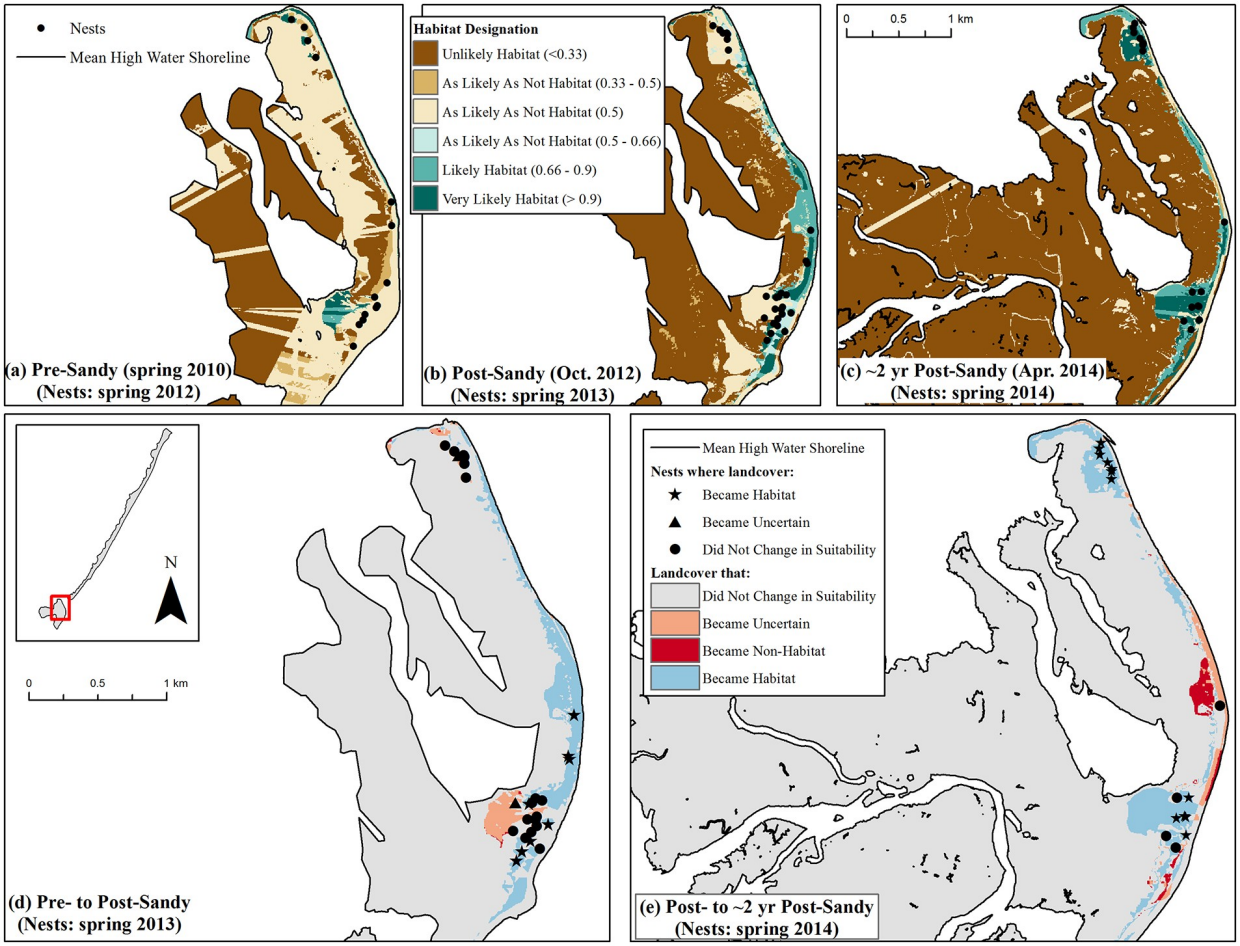
Examples of habitat change before Hurricane Sandy, immediately following Sandy, and ca. 2 years post-Sandy in a portion of the Little Beach Unit of the Edwin B. Forsythe National Wildlife Refuge on Pullen Island, New Jersey. Maps (a)-(c) show habitat in the year corresponding with the aerial imagery used for analysis, with piping plover nest points corresponding to the breeding season for which habitat was available^1^. Map (d) shows changes in habitat that ostensibly resulted from Hurricane Sandy, while map (e) shows changes in habitat since the hurricane occurred^2^. ^1^Nest points contributed by [67]. ^2^See footnotes accompanying Fig 4.

**Fig 7:**
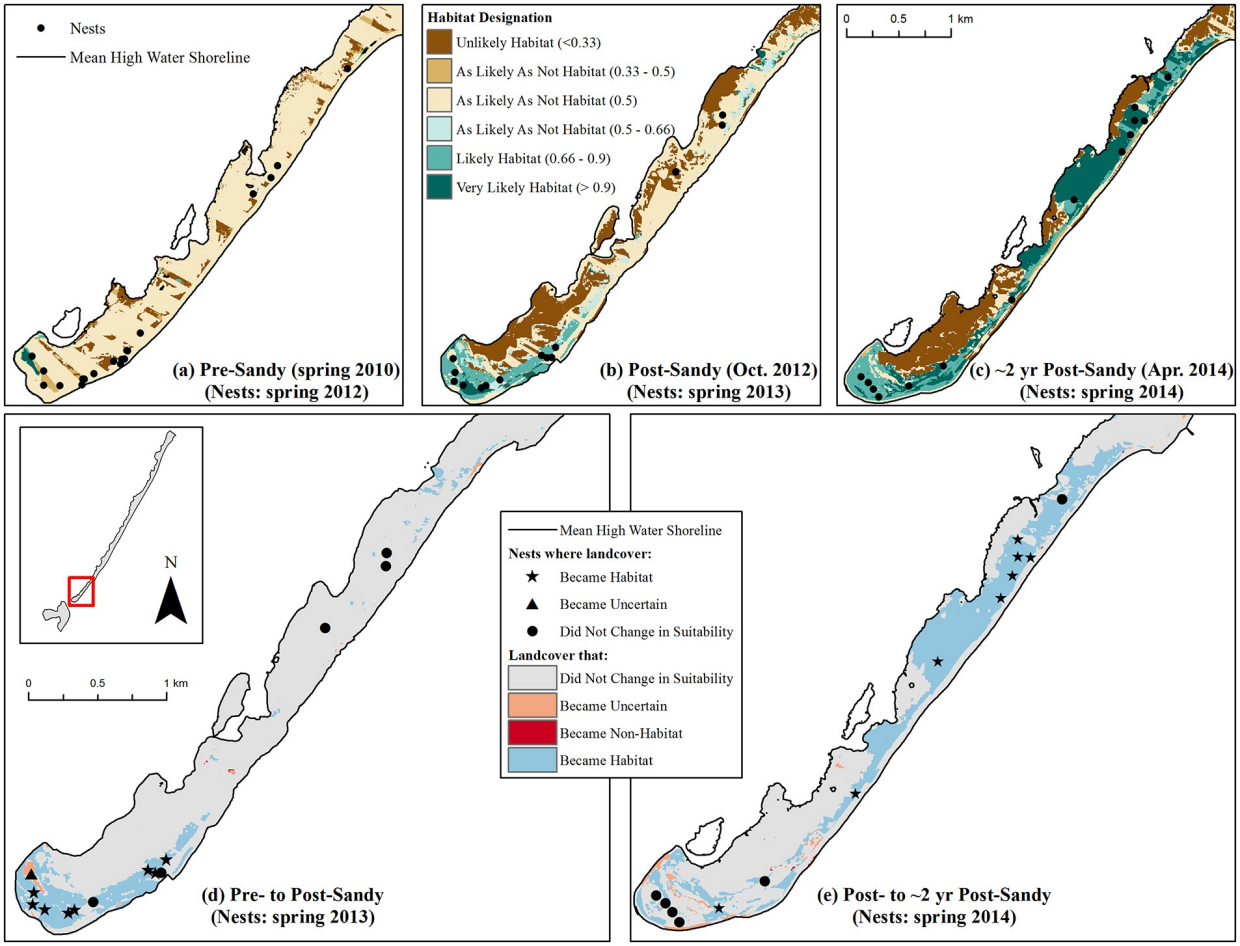
Examples of habitat change before Hurricane Sandy, immediately following Sandy, and ca. 2 years post-Sandy in the Holgate Unit of the Edwin B. Forsythe National Wildlife Refuge on Long Beach Island, New Jersey. Maps (a)-(c) show habitat in the year corresponding with the aerial imagery used for analysis, with piping plover nest points corresponding to the breeding season for which habitat was available^1^. Map (d) shows changes in habitat that ostensibly resulted from Hurricane Sandy, while map (e) shows changes in habitat since the hurricane occurred^2^. ^1^Nest points contributed by [67]. ^2^See footnotes accompanying Fig 4.

Both New Jersey sites also saw an increase in habitat area between the pre- and post-Sandy periods (Tables 2 and 3; Fig 3a), with Pullen Island experiencing a net gain of 0.3 km^2^ (e.g., Fig 6) and Long Beach Island experiencing a net gain of 0.5 km^2^ (e.g., Fig 7). Unlike the New York sites, both New Jersey sites continued to experience habitat gains through the ca. 2 years post-Sandy period (Tables 2 and 3; Fig 3a). Pullen Island gained another 0.2 km^2^ as Long Beach Island gained an additional 0.8 km^2^ of habitat (e.g., Figs 6 and 7). The continued increase in habitat through 2014 at these New Jersey sites may have been an artifact of the timing in which aerial imagery used in modeling was captured. According to 2012 imagery used to predict habitat on the two New Jersey sites (S2 Table), beaches within the undeveloped portions of the study areas were narrow and partially underwater in the days immediately following the storm, with swash bars that had not yet welded back on to the islands. Such areas were predicted to be uncertain or non-habitat by the Plover Habitat BN. Although swash bars may have welded onto the island and beaches may have dried to create nesting habitat in the days or weeks following the storm, habitat was not apparent in this analysis until the 2014 aerial imagery (S2 Table). Therefore, larger amounts of plover habitat could have been available for the 2013 breeding season, and habitat amounts may have declined by the 2014/2015 breeding seasons, as observed for Rockaway and Fire Island. However, a lack of imagery depicting conditions between November 2012 and 2014 makes it difficult to fully understand observed patterns on Pullen and Long Beach islands other than the fact that habitat increased above pre-Sandy levels between 2012 and 2014.

**Fig 8:**
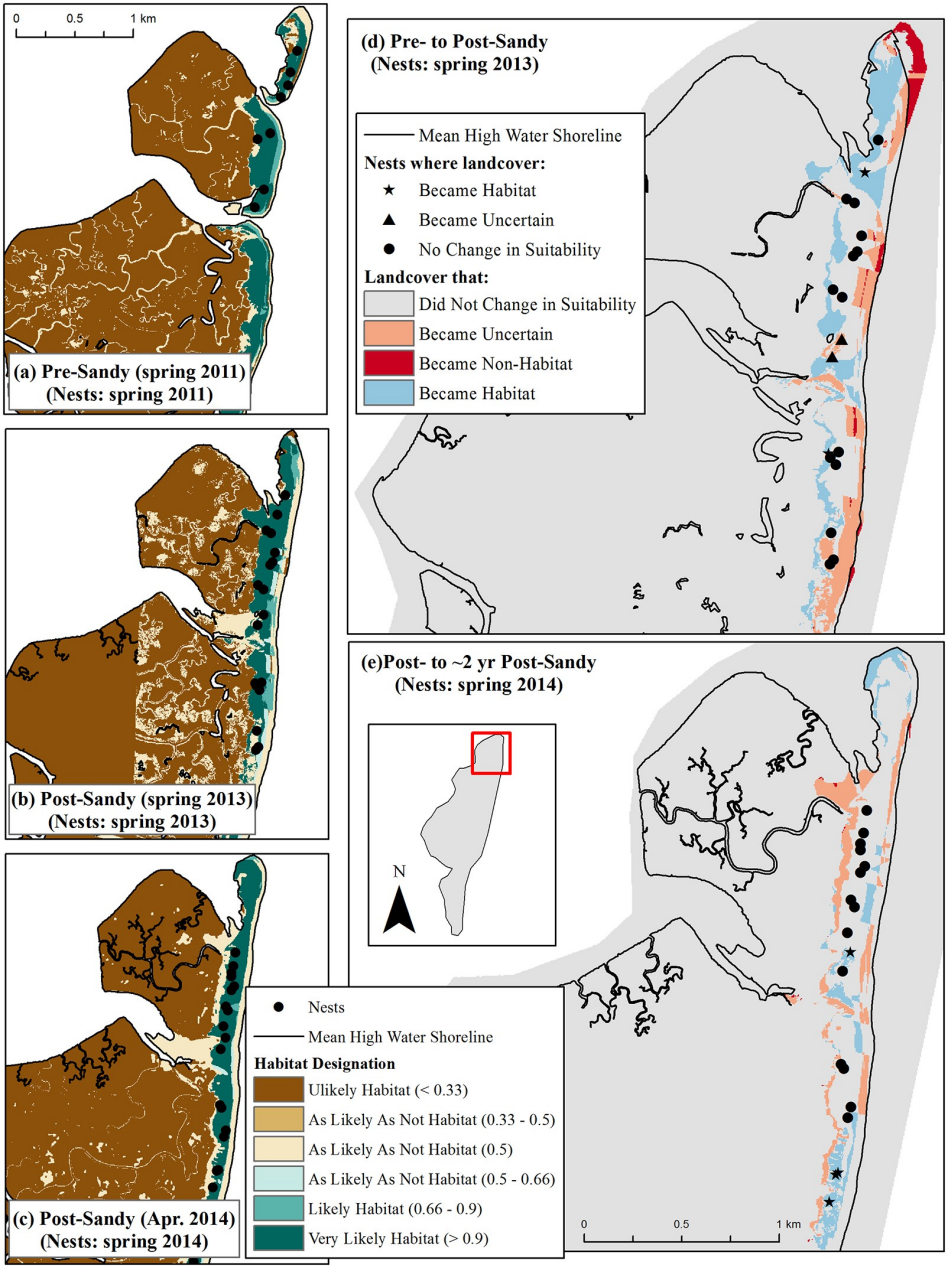
Examples of habitat change before Hurricane Sandy, immediately following Sandy, and ca. 2 years post-Sandy on Cedar Island, Virginia. Maps (a)-(c) show habitat in the year corresponding with the aerial imagery used for analysis, with piping plover nest points corresponding to the breeding season for which habitat was available^1^. Map (d) shows changes in habitat that ostensibly resulted from Hurricane Sandy, while map (e) shows changes in habitat since the hurricane occurred^2^. ^1^Nest points contributed by [69]. ^2^See footnotes accompanying Fig 4.

**Fig 9:**
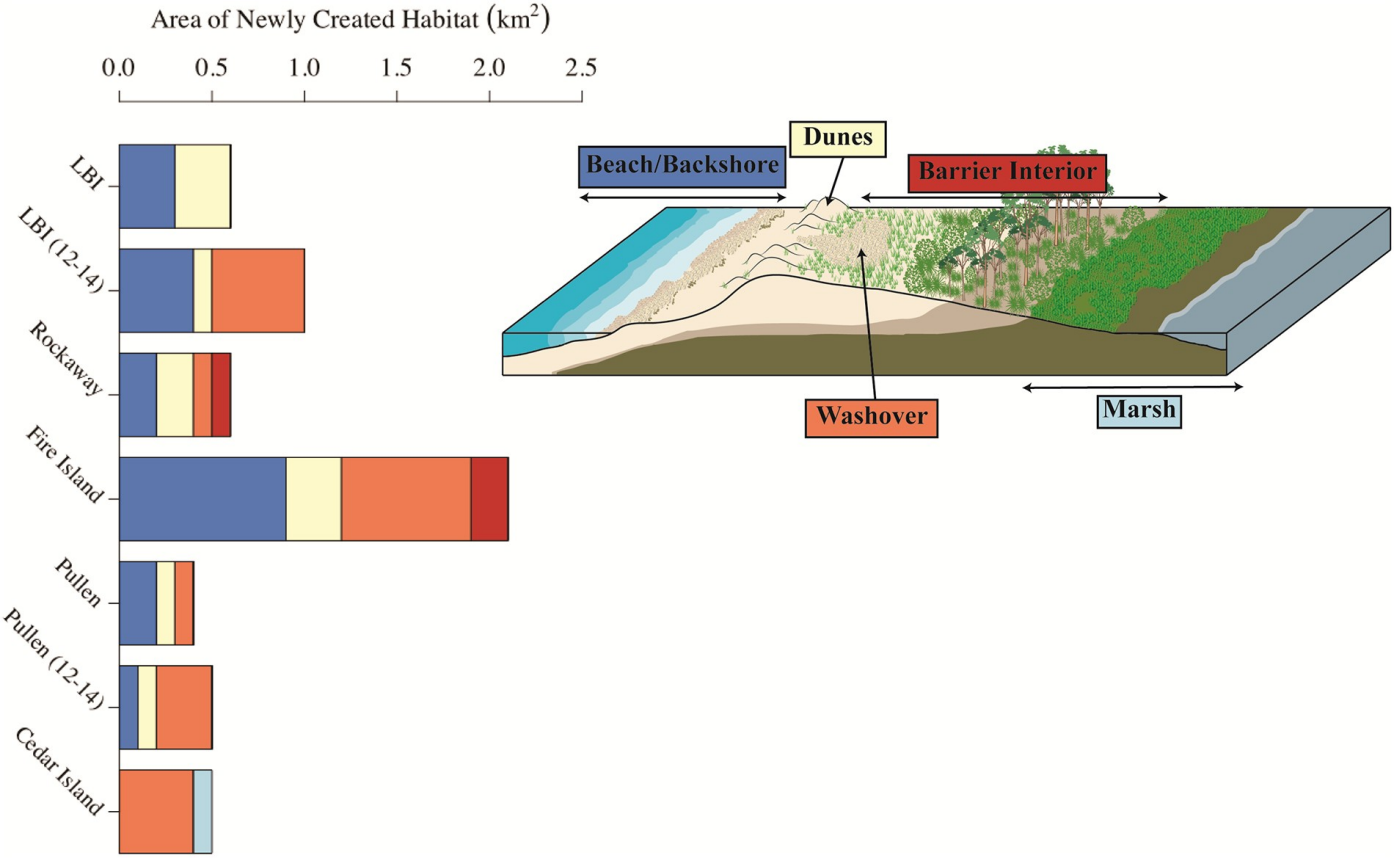
(a) Area of piping plover nesting habitat, according to predictions by the Plover Habitat Bayesian network, that was created immediately after Hurricane Sandy (i.e., newly created habitat between the pre- and post-Sandy periods). Bars showing habitat area are divided based on the geomorphic settings (defined in [34]) that were present after Hurricane Sandy according to October/November 2012 aerial photography and lidar. Because habitat continued to increase on Long Beach Island (LBI) and Pullen Island between the post- and ca. 2 years post-Sandy periods, we also include the amount and proportions of habitat created during that second time period for those sites (labeled with ‘12–14’ to denote the second period of habitat gain). Geomorphic settings for ‘12–14’ bars reflect settings available in the ca. post-Sandy period (June 2014). We combined the ‘beach’ and ‘backshore’ settings here for display purposes. Colors comprising bars in (a) are defined by colors of setting names in (b), and sites are listed from most to least developed.

Cedar Island was the only location that did not gain habitat after the storm. The island as a whole decreased in size between the pre-Sandy and ca. 2 years post-Sandy periods [47], and the amount of habitat similarly declined from 2.2 km^2^ (pre-Sandy) to 1.6 km^2^ (post-Sandy) to 1.4 km^2^ (ca. 2 years post-Sandy; Tables 2 and 3; Figs 3a and 8). However, where habitat was lost and gained could be of particular significance for this study area. Cedar Island lost 0.7 km^2^ of habitat in the beach/backshore while it gained 0.4 km^2^ of new habitat as overwash between the pre- and post-Sandy periods (Fig 9). An example of this can be seen in Fig 8, particularly in the interior strips of blue in panel (d) where new habitat was created in overwash pushing into what was marsh before the storm. Arguably lower quality habitat closer to the ocean was lost as higher quality overwash habitat [28] was gained, and overall habitat quality on Cedar Island may have stayed the same or improved even as the net amount of habitat declined. In addition, this study area was second only to Fire Island in the amount of habitat it supported despite being the smallest of the five study areas (Fig 3a).

It is unclear why the hurricane did not have a positive impact on the net amount of piping plover habitat on Cedar Island. This site was the farthest of the five sites from the epicenter of the storm, and the island may have been too far from the disturbance to create new early successional habitat. Alternatively, it is possible that annual, lower intensity disturbances are able to maintain large quantities of piping plover habitat on this undeveloped barrier island, and a single storm would not have had significant effects on already high habitat availability. Cedar Island is representative of low elevation islands that often occur in the absence of development and artificial stabilization, where a lack of a stable dune system allows for the prevalence of low-elevation overwash [75, 76] and maintenance of piping plover habitat. A storm like Hurricane Sandy may have more substantial impacts on early successional piping plover habitat on undeveloped, higher elevation islands with more stable dune structures [75, 76], as we observed with Pullen Island. Our results demonstrate that habitat response to storms involves complex interactions between prior geomorphology, habitat, and local storm conditions.

### Influence of human development on habitat creation

Although this storm increased habitat at the majority of our study sites, the location, amount, and longevity of that new early successional habitat appeared to be influenced by the level of human development or modification at the site. The percentage increase in the amount of habitat gained between the pre- and post-Sandy periods was inversely proportional to the amount of development in the study area for Rockaway, Fire Island, and Pullen Island. The densely developed Rockaway Peninsula saw the smallest increases in habitat (+9%), while moderately developed Fire Island (+116%) and undeveloped Pullen Island (+300%) saw much larger increases in habitat immediately following the storm (Table 3; Fig 3a).

The length of time habitat persisted after the storm also was inversely proportional to the amount of development on Rockaway, Fire Island, and Pullen Island. Habitat continued to be created through the ca. 2 years post-Sandy period on undeveloped Pullen Island, with a 50% increase in habitat between the post- and ca. 2 years post-Sandy periods. On moderately developed Fire Island, the amount of habitat declined by 17% between the post- and ca. 2 years post-Sandy periods; however, habitat area remained above pre-Sandy levels as of 2015. On the densely developed Rockaway Peninsula, habitat gained immediately after the storm did not persist through 2014, and habitat was below pre-Sandy levels by the ca. 2 years post-Sandy period (Table 3; Fig 3a).

The most developed site, Long Beach Island, and one of the undeveloped sites, Cedar Island, offered exceptions to these patterns. Hypotheses regarding why there was not a net increase in habitat on undeveloped Cedar Island are given in the previous section. Because Long Beach Island is highly developed and stabilized, we expected to see little change in habitat as a result of Hurricane Sandy. However, habitat increased at this location by 250% between the pre- and post-Sandy periods and continued to increase through the ca. 2 years post-Sandy period. Processes occurring in the low-elevation, undeveloped portion of the island, which is federally protected as the Holgate Unit of the Edwin B. Forsythe National Wildlife Refuge, drove these patterns. On Long Beach Island, 50% and 40% of all new habitat gained in the post-Sandy and ca. 2 years post-Sandy periods, respectively, occurred in this undeveloped area, which covers just 13% of the total study area (Fig 3a).

In addition to the amount and longevity of habitat created, development may have influenced the location of newly formed habitat. For all sites with some development, a disproportionately large amount of habitat, including newly created habitat following Hurricane Sandy, occurred in the undeveloped portions of the study areas. Although only 13% of Long Beach Island was considered undeveloped, 43% and 60% of all habitat was located in the undeveloped portion of the island that forms the Holgate Unit of the Edwin B. Forsythe National Wildlife Refuge during the post- and ca. 2 years post-Sandy periods, respectively (Fig 3a). Of all new habitat created immediately after the storm, 50% of that habitat formed in this undeveloped region between the pre- and post-Sandy periods, and another 40% of all new habitat that formed between the post- and ca. 2 years post-Sandy periods occurred in this region.

We observed similar patterns for the two developed New York sites. On the Rockaway Peninsula, 58–64% of all habitat in 2010–2014 was located along the undeveloped portion of the island in and adjacent to the Breezy Point Unit of the Gateway National Recreation Area, which only covers 27% of the study area (Fig 3a). Sixty-six percent of all habitat created immediately after the storm occurred in this undeveloped region. On Fire Island, where 69% of the study area was considered undeveloped, we found that 79–88% of all predicted habitat in 2011–2015 occurred in undeveloped stretches of the island (Fig 3a), and 81% of all habitat created immediately after the storm occurred in these undeveloped regions.

Development may have also influenced how habitat formed within discrete geomorphic settings. At our most developed site on Long Beach Island—where hotels, houses, and other infrastructure blocked overwash along much of the study area—all new habitat was gained equally within the beach/backshore and in dune complexes between the pre- and post-Sandy periods (Fig 9). Between the pre- and post-Sandy periods, habitat was also created in high proportions in the beach/backshore and in dune complexes on Rockaway (66% of all new habitat), Fire Island (57%), and Pullen Island (25%). However, new habitat was created in increasingly higher proportions in washovers as sites became less developed from Rockaway (17% of all new habitat in washover) and Fire Island (33%) to Pullen (44%) and Cedar islands (80%; Fig 9). Long Beach Island again offered an exception to this pattern, gaining 50% of new habitat in washover between the post- and ca. 2 years post-Sandy periods (Fig 9). However, the low-elevation, protected Holgate Unit was again responsible for these patterns; all new habitat gained in overwash occurred in this undeveloped region.

Some researchers suggest that overwash habitats are of highest quality for piping plover nesting due to their generally increased proximity to bayside foraging areas and greater distance from high energy shorelines [21, 28, 30]. The Plover Habitat BN and underlying supplemented iPlover dataset support this assertion; landcover combinations containing ‘Washover’ as the geomorphic setting have the highest probability of being habitat (0.77) over all other settings (0.13–0.70). Therefore, if we assume that washover habitat is of higher quality for piping plovers compared to habitat created in other geomorphic settings, this suggests that development influences both the net amount of habitat created as well as the quality of that new habitat.

Development and other human modifications are known to affect the geomorphic evolution of barrier islands. Sand fencing, hotels, homes and other human structures can alter aeolian transport and act as obstacles for the deposition of dune sediments and overwash [63, 64]. Even seemingly ‘natural’ structures, like dunes and berms intentionally constructed to add elevation along barrier islands, can restrict overwash processes and affect barrier island evolution [65]. Such changes to the way sand is transported in these systems, which can occur even at moderate levels of development [66], lead to higher rates of erosion and lower barrier islands that have a reduced capacity for recovery following storms [19, 63, 64, 66]. In addition, sand that overlays parking lots, roads, and other human structures as overwash is frequently removed as part of post-hurricane clean-up efforts, and these areas represent lost opportunities for new potential nesting habitat [20]. Therefore, it is not surprising that little habitat was created after Hurricane Sandy along the developed portions of our study sites. Piping plovers predominantly nest in overwash fans, backshore areas, and the swales of low-elevation dune complexes [31, 32, 34] on wide beaches [36, 77], and the creation of such areas would be minimized in developed areas that impede overwash and encourage erosion. This illustrates one mechanism by which human development and beach/dune stabilization are threats to coastal species like piping plovers [72].

As our results demonstrate, development may affect the capacity of storms to create early successional habitat. Although human development may not actually alter the intensity or frequency of coastal storms, it can alter the expression of storms by preventing, erasing, or minimizing storm impacts on barrier islands [78]. For example, high frequency, low intensity storms may be able to maintain large areas of early successional habitats on undeveloped islands like Cedar Island. However, more powerful storms that occur less frequently may be needed to create habitats on developed islands, like the Rockaway Peninsula and Fire Island. From the perspective of species like the piping plover, development may therefore appear to ‘lengthen’ the time between disturbances if only rarer, high intensity storms have the capacity to create habitat. Because ecosystems can be negatively impacted by alterations to natural disturbance regimes ([79], e.g., [80, 81]), particularly the lengthening of those regimes [82], development may indirectly threaten piping plovers and other early successional species by reducing storm-induced habitat creation and shortening habitat patch lifetimes. As observed here, the presence of multiple undeveloped barrier islands or long stretches of undeveloped shorelines on developed islands will be of critical importance for the persistence of early successional species like piping plovers in dynamic coastal environments.

**Fig 10:**
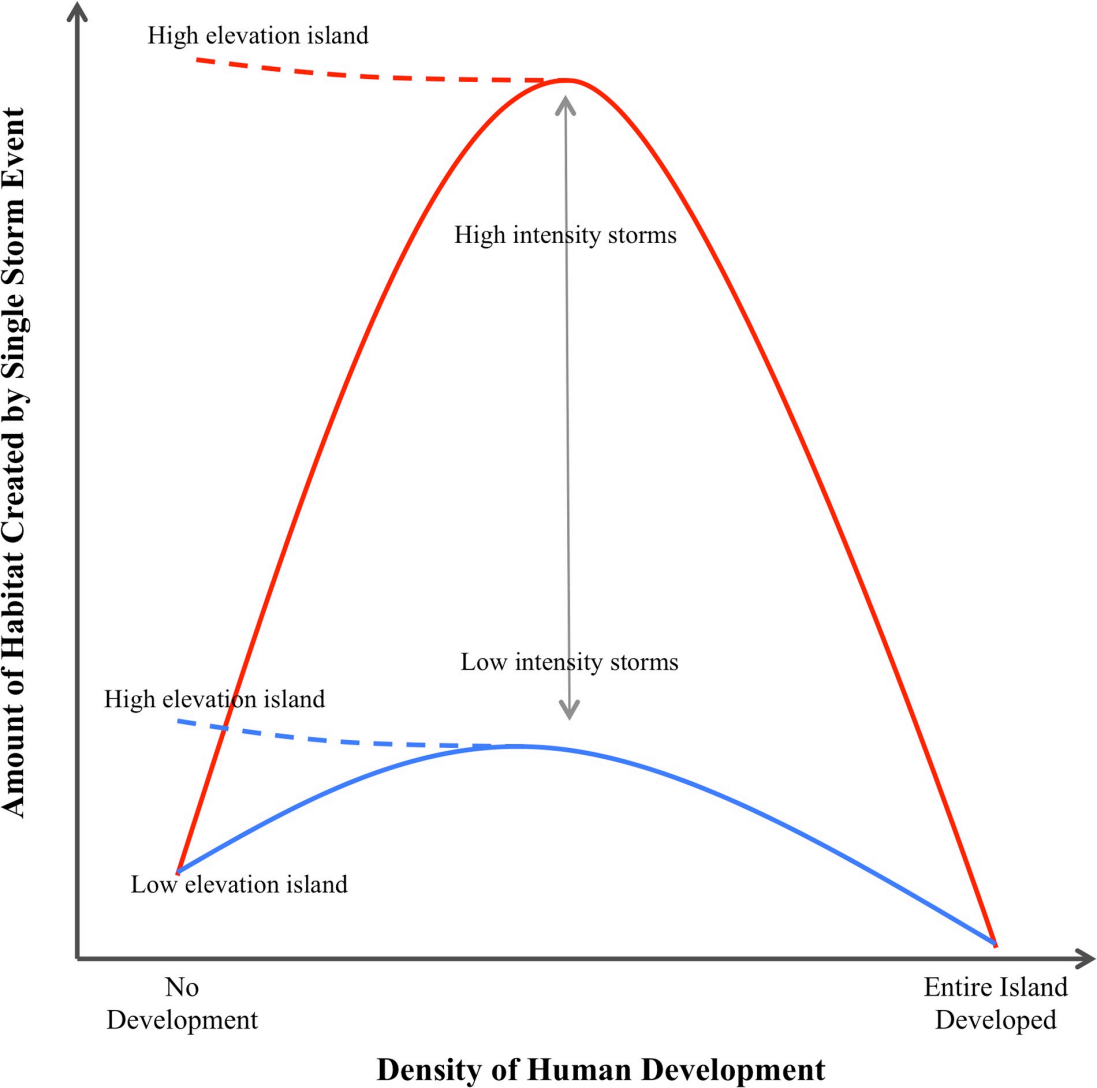
Proposed relationship between the density of human development across a barrier island and the amount of early successional habitat created by a single low intensity (blue line) or high intensity (red line) storm event. In this relationship, no single storm event will have a major impact on the amount of early successional habitat on low elevation, undeveloped islands because smaller intensity storms maintain maximum amounts of habitat through time. A single storm will also have a minor impact on habitat on islands with a high density of human development because shoreline modifications prevent overwash and encourage erosion, minimizing the ‘opportunity space’ for new habitats to be created. On high elevation, undeveloped islands (dashed lines) or on islands with moderate levels of development (middle of x-axis), less frequent high-intensity storms are needed to create overwash and new habitats; however, undeveloped stretches of shoreline provide opportunities for habitat to be created. As a result, a large storm like Hurricane Sandy can have a major impact in the amount of habitat available.

Therefore, we suggest the possibility of parabolic relationships between storm-induced changes in habitat and the density of human development (Fig 10). In this relationship, no single storm event will have a major impact on the amount of habitat available for nesting piping plovers (or other early successional coastal species) on undeveloped, unstabilized, low elevation sites [76] as smaller intensity, higher frequency storms are able to maintain large amounts of habitat through time. These islands essentially reach a saturation point in habitat that is maintained through time. At the other end of the development spectrum, even very large storms may again only have a minor impact on the amount of habitat created because development directly and indirectly prevents overwash and encourages erosion [19, 63, 64, 66], thus minimizing the ‘opportunity space’ for new habitats to be created. For high elevation, undeveloped islands as well as islands in the middle of the development spectrum—where swaths of undeveloped shoreline are present in juxtaposition with areas of heavy human development—larger, less frequent storms are needed to create overwash and new habitats. However, undeveloped stretches of shoreline provide opportunities for habitat to be created. As a result, a large storm like Hurricane Sandy can have a major impact in the amount of habitat available at high elevation, undeveloped or on moderately developed sites (Fig 10). In addition, storm intensity will drive the absolute change in habitat on this spectrum of island types, with larger storms able to create more overwash and piping plover habitat compared to less powerful storms (Fig 10).

### Population-level responses to changes in piping plover habitat

Based on data on nest locations, population size, and population productivity collected by collaborators in New York [68], New Jersey [67] and Virginia [69], piping plovers appear to have responded to the increase in habitat following Hurricane Sandy at the population level. This species exhibits strong site fidelity, often nesting within 200 m of the same location in successive years [83, 84]. However, a large portion of nesting pairs exploited new habitats in the breeding season immediately following Hurricane Sandy on Long Beach Island (57% of all nests in new habitat), the Rockaway Peninsula (52%), Pullen Island (36%), and Cedar Island (14%; no data for Fire Island; Table 3). On the New Jersey sites—where habitat continued to increase through the ca. 2 years post-Sandy period—a high percentage of nests were also established in new habitat in the 2014 breeding season, with 57% and 65% of all nests occurring in newly created habitat on Long Beach and Pullen islands, respectively (Table 3). In addition, birds continued to colonize newly formed habitats in this period on the remaining 3 study areas despite net habitat declines; 23%, 36%, and 25% of all nests occurred in newly created habitats ca. 2 years post-Sandy on Rockaway, Fire Island, and Cedar Island, respectively (Table 3).

The strength of site fidelity in this species became clearer where nests were established in the ca. 2 years post-Sandy breeding season. The percentage of nests established on landcover that did not change in suitability between the post- and ca. 2 years post-Sandy periods increased from the previous period for all sites except for Pullen Island, where habitat continued to form through 2014 (Table 3). There was also an increasing number of nests in locations where the model predicted a shift from habitat (post-Sandy) to non-habitat (ca. 2 years post-Sandy) on the Rockaway Peninsula (12% of nests) and Fire Island (10.5%; Table 2). Site fidelity, even as habitat quality declines, is expected for species in dynamic, unpredictable habitats [85]. Our results suggest that site fidelity could cause piping plovers to nest in landcover that is of poor quality in the absence of new disturbances—landcover that could promote a population sink [86] with mortality exceeding reproduction. When development precludes the creation or accelerates the degradation of early successional habitats, birds may nest in non-habitat in increasing numbers, which could lead to population declines for the species. A similar dynamic was observed by Anteau et al. [87] for a subpopulation of piping plovers on Lake Sakakawea, North Dakota.

Population size and average productivity also increased following increases in post-Sandy habitat. Average productivity increased at all sites from the pre-Sandy (spring 2012) breeding season, where average productivity ranged from 0.3–0.9 chicks fledged per breeding pair, to the ca. 2 years post-Sandy (spring 2014) breeding season, where productivity ranged from 1.0–2.4 chicks fledged per breeding pair (Fig 3c). With average productivity rates ranging from 1.1–1.6 chicks fledged per pair, productivity has remained above pre-Sandy levels as of the spring 2015 breeding season for all study areas except Fire Island (Fig 3c).

As also observed by Bourque et al. [88], we found that the relationship between the storm-induced increase in habitat and piping plover population size was less clear. Population sizes, measured as the number of breeding pairs, remained steady or declined from the pre-Sandy (spring 2012) through the ca. 2 years post-Sandy (spring 2014) breeding seasons at all sites except for Pullen Island (Fig 3b). However, populations—with the exception of that on Fire Island—have increased between the 2014 and 2015 breeding seasons (Fig 3b). Although other factors beyond habitat availability (e.g., predation rates, management activities, human disturbance, regional productivity) influence populations, the ultimate growth in population size and productivity suggests that new habitat created by Hurricane Sandy had a positive impact on this species at the population-level following a 2 to 3-year time lag.

## Conclusions

In this study, we demonstrated an ability to quantify the integrated response of piping plover habitat over a range of geographic locations through time. This capability is important to understanding the net ecological response to storms and longer-term processes. Here, Hurricane Sandy increased levels of nesting habitat, and piping plovers responded by exploiting newly created habitats in the breeding season immediately following the storm—which was ultimately associated with increased population productivity and abundance levels. However, the amount, longevity, and location of newly created habitat appeared to be inversely related to the amount of human development on study sites. Our results quantify the importance of storms in creating and maintaining coastal habitats for beach-nesting species like piping plovers, and these results suggest a negative correlation between human development and beneficial ecological impacts of these natural disturbances.

## Supporting information

S1 File Accuracy and sensitivity testing of the Bayesian network and thresholds used for analysis. (DOCX)

S1 Fig The receiver operating characteristic (ROC) curve for the Plover Habitat Bayesian network (BN). This method plots the percentage of true positives (‘sensitivity’) as a function of percentage false positives (‘1-specificity’) over the continuum of probability thresholds, and the area under the ROC curve (AUC) is a measure of overall network performance. A top-performing network will have a ROC curve that falls into the top left portion of the plot and an AUC approaching 1.
(DOCX)

S1 Table Error rate of the Plover Habitat Bayesian network reported as percentage error rate resulting from 10-fold cross-validation, using the node for ‘Habitat Designation’ as the target variable for testing. We also evaluated the sensitivity of the model to selected parameters by systematically removing nodes.
(DOCX)

S2 Table Remotely sensed lidar and aerial photography used to characterize study areas and iPlover dataset points. Information derived from these data sources included distance to ocean, distance to foraging areas, beach width, and elevation for each study area prior to Hurricane Sandy (2010/2011), immediately after the storm (2012), and ca. 2 years after the storm (2014/2015). Light gray cells highlight data used for pre-Sandy analyses, medium gray cells highlight data used for post-Sandy analyses, and dark gray cells highlight data used for ca. 2 years post-Sandy analyses.
(DOCX)

S3 Table Effectiveness of the Intergovernmental Panel on Climate Change’s (IPCC’s) likelihood scale [8] for defining habitat predictions made by the Plover Habitat Bayesian network (BN). Under this scale, a combination of landscape characteristics was considered ‘likely habitat’ if it was associated with a BN probability ≥ 0.66, ‘uncertain’ with a probability 0.33–0.66, and ‘unlikely habitat’ with a probability ≤ 0.33. We assumed that the IPCC scale accurately delineated habitat if the landscape characteristics associated with the majority of piping plover nest points had a probability ≥ 0.66 of being habitat and if the characteristics associated with the majority of random points had a probability ≤ 0.33 of being habitat.
(DOCX)
