# Supplementary material for: Using a Bayesian network to understand the importance of coastal storms and undeveloped landscapes for the creation and maintenance of early successional habitat
Source: PLoS One. 2019 Jul 25;14(7):e0209986. doi: 10.1371/journal.pone.0209986 (PMC6657824; doi:10.1371/journal.pone.0209986)
Supplement: S1 File — (DOCX) [file pone.0209986.s001.docx]

**S1 File. Accuracy and sensitivity testing of the Bayesian network and thresholds used for analysis.**

**Network validation, accuracy, and sensitivity**

Testing and validating a Bayesian network (BN) is an essential step in the model-building process [1]. We examined the accuracy of the Plover Habitat BN (depicted in Fig 2) using two different approaches: (1) *k*-fold cross-validation and (2) analysis of the receiver operating characteristic (ROC) curve. Statistical evaluation was conducted in MatLab (version 9.5; MathWorks).

***k*-fold cross-validation – methods and results**

We examined the BN’s skill at predicting piping plover habitat through *k*-fold cross-validation in the Python module CVNetica according to Fienen and Plant [2] using the supplemented iPlover dataset. We utilized 10 folds and calculated classification error for the output node ‘Habitat Designation.’ Under the 10-fold cross-validation procedure, the supplemented iPlover dataset was randomly divided into ten groups, with each tenth representing a ‘fold’. The model was iteratively trained on data from 9 of the 10 folds (90% of the data), and model predictions were tested against data in (*i*) the same training folds to calculate calibration error and (*ii*) the fold that was left out of the training data (10% of the data) to calculate validation error. Calibration and validation error rates for each of the ten combinations of testing and training data were averaged to calculate mean ensemble error rates. For ease of testing, we utilized a threshold of 0.5 to separate ‘Habitat’ from ‘Non-Habitat’. For instance, if the BN predicted that that the combination of landscape variables associated with an actual nest point had a probability of being habitat > 0.5, this counted as a correct prediction (or a ‘true positive’). If the BN predicted that a combination of variables associated with a nest point had a probability of being habitat < 0.5, this counted as an incorrect prediction (or false negative) resulting in error.

To evaluate the sensitivity of the network to individual nodes, we repeated the 10-fold cross-validation procedure as described in the previous paragraph, removing a single node each time. We recalculated network error rate with each node-removal and compared that rate to the error rate of the full model. Large changes in error rate compared to that of the full model indicated that the network was sensitive to the node that had been removed in that iteration.

From this analysis, we found a mean error rate of 0.05 during the calibration phase of testing and 0.23 during the validation phase of testing for the full network displayed in Fig 2 (S1 Table). Although the removal of any one node did not result in major changes in the network’s predictive accuracy, the removal of Beach Width resulted in the largest change in error rate (mean accuracy of 0.18). The removal of Distance to Ocean, Distance to Foraging, and Elevation resulted in slightly higher accuracy (0.22); while the removal of Vegetation Density (0.25), Geomorphic Setting (0.24), and Substrate Type (0.24) resulted in slightly lower accuracy (S1 Table). The loss of the node for Vegetation Type did not change accuracy compared to the full network (0.23; S1 Table). Because network accuracy did not substantially change with the removal of specific nodes, we elected to maintain all nodes to generalize model utility for additional applications. The Plover Habitat BN was developed as part of a broader research program in which we model piping plover habitat over a regional scale (from Maine to North Carolina) and forecast habitat given sea-level rise [3]. Part of a BN’s utility lies in its ability to facilitate learning about causal relationships, to operate even when missing data, and to make predictions for more than one node (or characteristic) of interest [4]. This utility, particularly with regards to inferential power, relies on the nature, number, and redundancy of connections among nodes. The use of additional nodes for Beach Width, Elevation, Distance to Ocean, and Distance to Foraging also allows for stronger, redundant connections to additional BNs for shoreline change [5] and barrier island geomorphology [6] as well as flexibility in the characteristics of future barrier island states that we ultimately predict beyond the Habitat Designation node in the Plover Habitat BN.

**ROC curve – methods and results**

The ROC curve can be used to assess a network’s predictive accuracy over the continuum of prediction possibilities (here, 0 to 1) for models with a binary output [7]. Accuracy is plotted over a continuum of thresholds as opposed to a single, arbitrary threshold (such as 0.5 used above). This method plots the percentage of true positives (‘sensitivity’) as a function of percentage false positives (‘1-specificity’; see S1 Fig). The area under the ROC curve (AUC) is a measure of overall network performance across the continuum of prediction thresholds. A network that produces random predictions, which offers no classifier information, will have a ROC curve that falls along a 45^o^ line on the ‘sensitivity vs. 1-specificity’ plot (e.g., S1 Fig) and an AUC = 0.5. A top-performing network will have a ROC curve that falls into the top left portion of the plot and an AUC approaching 1. A network that consistently produces wrong predictions will have a ROC curve that falls into the bottom right portion of the plot and an AUC < 0.5 [7].

In our analysis, the ROC graph shows the increase in correct habitat classifications, at the expense of incorrectly classifying non-habitat. That is, with a sufficiently low probability threshold for selecting suitable habitat, every location would be classified as suitable, and there would be no false-positive classifications but many false negatives (e.g., the upper right point in the graph). Likewise, if a sufficiently high probability threshold were used, there would be no false-negatives, but not many locations would be classified as suitable. The ROC curve for our network was stationed in the upper left area of the plot (S1 Fig) with an AUC = 0.90 (or a < 10% false-negative classification rate), suggesting high predictive skill.

**Probabilistic suitability threshold performance**

To define and map what constituted ‘habitat’ vs. ‘non-habitat’ in spatial analyses, we were required to set thresholds for which probabilities indicated the presence of habitat versus non-habitat. We opted to use thresholds based on the Intergovernmental Panel on Climate Change’s (IPCC’s) likelihood scale [8]. Under this scale, a landscape cell was considered ‘very likely habitat’ if it had a probability > 0.90 of being habitat, ‘likely habitat’ with a probability 0.66–0.90, ‘uncertain’ with a probability 0.33–0.66, and ‘unlikely habitat’ with a probability < 0.33. Landscape cells with probabilities > 0.66 were considered habitat, while cells with probabilities < 0.33 were considered non-habitat. We examined whether these thresholds proved meaningful for identifying the likelihood of actual piping plover nesting. We determined the number of nests (289 total) where the associated combination of landscape characteristics was predicted to be habitat by the BN with a probability > 0.66 as well as the number of random points (278 total) where the associated combination of landscape characteristics was predicted to be habitat by the BN with a probability < 0.33.

We found that the Plover Habitat BN’s predictions for combinations of landscape characteristics associated with nests fell within the IPCC-derived threshold for habitat (*p* > 0.66) in 98% of cases. Similarly, predictions for combinations of landscape characteristics associated with random points fell within the IPCC-derived threshold for non-habitat (*p* < 0.33) in 85% of cases (S2 Table). These results support the utility of the IPCC likelihood thresholds in delineating habitat.

**Summary and conclusions**

We conducted two separate validation exercises to test the predictive accuracy of the BN used in our analysis. In the first analysis, we used 10-fold cross-validation, which maintains separate training and testing datasets and uses a single probability threshold (0.50) to delineate accurate predictions from predictive errors. The network’s mean validation error rate according to this analysis was 0.23. In the second analysis, we evaluated the BN’s ROC curve, which demonstrates a network’s predictive accuracy across a continuum of probability thresholds. We observed that the ROC curve was plotted in the upper left portion of the plot (S1 Fig) with an AUC = 0.90 (or an error rate < 10%). Furthermore, sensitivity analysis revealed that the BN was not substantially sensitive to any one node included in the network. Therefore, these analyses demonstrate that the Plover Habitat BN, as depicted in Fig 2, behaved with a high level of accuracy and is capable of providing robust predictions regarding the suitability of landcover as piping plover habitat.

In addition, we tested the validity of using the IPCC’s likelihood scale to determine appropriate probability thresholds for defining piping plover habitat from non-habitat. This analysis showed that the network assigned a probability > 66% to landcover characteristics associated with piping plover nests in 98% of the test cases. Likewise, the network assigned a probability < 33% to landcover characteristics associated with random points in 85% of the test cases. Therefore, we concluded that the IPCC likelihood thresholds could be used satisfactorily to delineate habitat.

**References**

1. Marcot B, Steventon J, Sutherland G, McCann R. Guidelines for developing and updating Bayesian belief networks applied to ecological modeling and conservation. Canadian Journal of Forest Research. 2006;36:3063-74.

2. Fienen M, Plant N. A cross-validation package driving Netica with python. Environmental Modelling & Software. 2015;63:14-23.

3. Zeigler S, Thieler E, Gutierrez B, Plant N, Hines M, Fraser J, et al. Smartphone technologies and Bayesian networks to assess shorebird habitat selection. DOI: 10.1002/wsb.820. Wildlife Society Bulletin

. 2017;41(4):666-77.

4. Uusitalo L. Advantages and challenges of Bayesian networks in environmental modelling. Ecological Modelling. 2007;303:312-8.

5. Gutierrez B, Plant N, Pendleton E, Thieler E. Using a Bayesian network to predict shoreline-change vulnerability to sea-level rise for the coasts of the United States. United States Geological Survey open file report 2014-1083.

<https://doi.org/10.3133/ofr201410832014>. 26 p.

6. Gutierrez B, Plant N, Thieler E, Turecek A. Using a Bayesian network to predict barrier island geomorphologic characteristics. Journal of Geophysical Research: Earth Surface. 2015;120:2451-75.

7. Pepe M. Receiver operating characteristic methodology. Journal of the American Statistical Association. 2000;95(449):308-11.

8. Intergovernmental Panel on Climate Change (IPCC), editor. Climate change 2014: Synthesis report. Contribution of working groups I, II, and III to the Fifth Assessment Report of the Intergovernmental Panel on Climate Change. Geneva, Switzerland: IPCC; 2014.
