# Supplementary material for: Using a Bayesian network to understand the importance of coastal storms and undeveloped landscapes for the creation and maintenance of early successional habitat
Source: PLoS One. 2019 Jul 25;14(7):e0209986. doi: 10.1371/journal.pone.0209986 (PMC6657824; doi:10.1371/journal.pone.0209986)
Supplement: S1 Fig — This method plots the percentage of true positives (‘sensitivity’) as a function of percentage false positives (‘1-specificity’) over the continuum of probability thresholds, and the area under the ROC curve (AUC) is a measure of overall network performance. A top-performing network will have a ROC curve that falls into the top left portion of the plot and an AUC approaching 1. (DOCX) [file pone.0209986.s002.docx]

S1 Fig. The receiver operating characteristic (ROC) curve for the Plover Habitat Bayesian network (BN). This method plots the percentage of true positives (‘sensitivity’) as a function of percentage false positives (‘1-specificity’) over the continuum of probability thresholds, and the area under the ROC curve (AUC) is a measure of overall network performance. A top-performing network will have a ROC curve that falls into the top left portion of the plot and an AUC approaching 1.
