# Supplementary material for: Using a Bayesian network to understand the importance of coastal storms and undeveloped landscapes for the creation and maintenance of early successional habitat
Source: PLoS One. 2019 Jul 25;14(7):e0209986. doi: 10.1371/journal.pone.0209986 (PMC6657824; doi:10.1371/journal.pone.0209986)
Supplement: S1 Table — We also evaluated the sensitivity of the model to selected parameters by systematically removing nodes. (DOCX) [file pone.0209986.s003.docx]

S1 Table. Error rate of the Plover Habitat Bayesian network reported as percentage error rate resulting from 10-fold cross-validation, using the node for ‘Habitat Designation’ as the target variable for testing. We also evaluated the sensitivity of the model to selected parameters by systematically removing nodes.

| **Node Removed** |  | **Mean Error Rate by Fold** | | | | | | | | | |  |
| --- | --- | --- | --- | --- | --- | --- | --- | --- | --- | --- | --- | --- |
|  |  | ***1*** | ***2*** | ***3*** | ***4*** | ***5*** | ***6*** | ***7*** | ***8*** | ***9*** | ***10*** | ***Mean*** |
| Full Model  (no nodes removed) | Calibration | 0.05 | 0.05 | 0.06 | 0.05 | 0.06 | 0.05 | 0.05 | 0.05 | 0.05 | 0.05 | 0.05 |
|  | Validation | 0.29 | 0.22 | 0.25 | 0.25 | 0.24 | 0.18 | 0.31 | 0.22 | 0.20 | 0.18 | 0.23 |
|  |  |  |  |  |  |  |  |  |  |  |  |  |
| Beach Width | Calibration | 0.05 | 0.05 | 0.06 | 0.05 | 0.05 | 0.06 | 0.06 | 0.06 | 0.06 | 0.06 | 0.06 |
|  | Validation | 0.29 | 0.14 | 0.18 | 0.22 | 0.22 | 0.16 | 0.25 | 0.22 | 0.08 | 0.09 | 0.18 |
| Elevation | Calibration | 0.07 | 0.07 | 0.07 | 0.07 | 0.07 | 0.07 | 0.06 | 0.08 | 0.07 | 0.07 | 0.07 |
|  | Validation | 0.18 | 0.25 | 0.27 | 0.20 | 0.18 | 0.29 | 0.31 | 0.22 | 0.18 | 0.14 | 0.22 |
| Distance to Ocean | Calibration | 0.06 | 0.06 | 0.06 | 0.06 | 0.07 | 0.06 | 0.05 | 0.06 | 0.06 | 0.06 | 0.06 |
|  | Validation | 0.25 | 0.12 | 0.27 | 0.31 | 0.18 | 0.24 | 0.29 | 0.16 | 0.14 | 0.21 | 0.22 |
| Distance to Foraging | Calibration | 0.08 | 0.08 | 0.08 | 0.07 | 0.08 | 0.08 | 0.08 | 0.07 | 0.08 | 0.08 | 0.08 |
|  | Validation | 0.27 | 0.24 | 0.29 | 0.24 | 0.20 | 0.16 | 0.20 | 0.29 | 0.16 | 0.16 | 0.22 |
| Geomorphic Setting | Calibration | 0.08 | 0.08 | 0.07 | 0.07 | 0.07 | 0.08 | 0.08 | 0.08 | 0.07 | 0.07 | 0.07 |
|  | Validation | 0.25 | 0.25 | 0.33 | 0.24 | 0.24 | 0.14 | 0.16 | 0.22 | 0.31 | 0.21 | 0.24 |
| Substrate Type | Calibration | 0.07 | 0.07 | 0.07 | 0.08 | 0.07 | 0.08 | 0.08 | 0.08 | 0.08 | 0.07 | 0.07 |
|  | Validation | 0.25 | 0.20 | 0.31 | 0.24 | 0.33 | 0.16 | 0.20 | 0.24 | 0.20 | 0.27 | 0.24 |
| Vegetation Type | Calibration | 0.06 | 0.06 | 0.05 | 0.06 | 0.06 | 0.06 | 0.06 | 0.06 | 0.05 | 0.06 | 0.06 |
|  | Validation | 0.20 | 0.24 | 0.20 | 0.29 | 0.16 | 0.22 | 0.20 | 0.25 | 0.25 | 0.27 | 0.23 |
| Vegetation Density | Calibration | 0.07 | 0.07 | 0.08 | 0.08 | 0.08 | 0.07 | 0.07 | 0.08 | 0.07 | 0.07 | 0.07 |
|  | Validation | 0.20 | 0.27 | 0.25 | 0.24 | 0.29 | 0.16 | 0.24 | 0.27 | 0.31 | 0.29 | 0.25 |
