# Supplementary material for: Using a Bayesian network to understand the importance of coastal storms and undeveloped landscapes for the creation and maintenance of early successional habitat
Source: PLoS One. 2019 Jul 25;14(7):e0209986. doi: 10.1371/journal.pone.0209986 (PMC6657824; doi:10.1371/journal.pone.0209986)
Supplement: S2 Table — Information derived from these data sources included distance to ocean, distance to foraging areas, beach width, and elevation for each study area prior to Hurricane Sandy (2010/2011), immediately after the storm (2012), and ca. 2 years after the storm (2014/2015). Light gray cells highlight data used for pre-Sandy analyses, medium gray cells highlight data used for post-Sandy analyses, and dark gray cells highlight data used for ca. 2 years post-Sandy analyses. (DOCX) [file pone.0209986.s004.docx]

S2 Table. Remotely sensed lidar and aerial photography used to characterize study areas and iPlover dataset points. Information derived from these data sources included distance to ocean, distance to foraging areas, beach width, and elevation for each study area prior to Hurricane Sandy (2010/2011), immediately after the storm (2012), and ca. 2 years after the storm (2014/2015). Light gray cells highlight data used for pre-Sandy analyses, medium gray cells highlight data used for post-Sandy analyses, and dark gray cells highlight data used for ca. 2 years post-Sandy analyses.

| **Study Area** | **Dataset** | **Date of Acquisition** | **Original Resolution** | **Source and Availability** |
| --- | --- | --- | --- | --- |
| Fire Island | 2011 National Oceanic and Atmospheric Administration (NOAA) Ortho-rectified Color Mosaic of Fire Island, New York | 25 Oct 2011 | 0.5 m | https://coast.noaa.gov/digitalcoast |
|  | United States Department of Agriculture (USDA)-Farm Service Agency (FSA)- Aerial Photography Field Office (APFO) National Agriculture Imagery Program (NAIP) MrSID Mosaic (used for West Hampton Dunes only) | Oct 2011 | 1 m | https://gdg.sc.egov.usda.gov/ |
|  | 2010 U.S. Army Corps of Engineers (USACE) National Coastal Mapping Program (NCMP) Topobathy Lidar: Atlantic Coast (NY) | 19-27 Aug 2010 | 2 m | https://coast.noaa.gov/digitalcoast |
|  | Hurricane Sandy: Rapid Response Imagery of the Surrounding Regions | 31 Oct to 06 Nov 2012 | 0.35 m | http://ngs.woc.noaa.gov/storms/sandy |
|  | 2012 U.S. Geological Survey (USGS) topographic lidar: Northeast Atlantic Coast Post-Hurricane Sandy | 5-29 Nov 2012 | 1 m | https://coast.noaa.gov/digitalcoast |
|  | 2015 Fire Island Ortho-Imagery (collected at request of Virginia Tech) | 15 Apr 2015 | 0.15 m | Available upon request from Dr. James Fraser (fraser@vt.edu) |
|  | 2014 NOAA Post-Sandy Topobathymetric LiDAR: Void DEMs South Carolina to New York | Nov 2013 to Jun 2014 | 1 m | https://coast.noaa.gov/digitalcoast |
| Rockaway Peninsula | USDA-FSA-APFO NAIP MrSID Mosaic | Oct 2011 | 1 m | https://gdg.sc.egov.usda.gov/ |
|  | 2010 USACE NCMP Topobathy Lidar: Atlantic Coast (NY) | 19-27 Aug 2010 | 2 m | https://coast.noaa.gov/digitalcoast |
|  | Hurricane Sandy: Rapid Response Imagery of the Surrounding Regions | 3-4 Nov 2012 | 0.35 m | http://ngs.woc.noaa.gov/storms/sandy |
|  | 2012 USACE Topobathy Lidar: Post Sandy (NJ & NY) | 16 Nov 2012 | 1 m | https://coast.noaa.gov/digitalcoast |
|  | 2014 NOAA Ortho-rectified Mosaic of Hurricane Sandy Coastal Impact Area | 1 Jan to 21 Apr 2014 | 0.35 m | https://coast.noaa.gov/digitalcoast |
|  | 2013–2014 USGS Coastal and Marine Geology Program (CMGP) LiDAR: Post Sandy (New York City) | 6 Aug 2013 to 21 Apr 2014 | 0.7 m | https://coast.noaa.gov/digitalcoast |
| Long Beach and Pullen islands | USDA-FSA-APFO NAIP MrSID Mosaic | ‘leaf-on’ months 2010 | 1 m | https://gdg.sc.egov.usda.gov/ |
|  | 2010 USACE Joint Airborne Lidar Bathymetry Technical Center of eXpertise (JALBTCX) Lidar: New Jersey (Topo) | 28 Aug to 11 Sept 2010 | 2 m | https://coast.noaa.gov/digitalcoast |
|  | Hurricane Sandy: Rapid Response Imagery of the Surrounding Regions | 31 Oct to 06 Nov 2012 | 0.35 m | http://ngs.woc.noaa.gov/storms/sandy |
|  | 2012 USGS EAARL-B Coastal Topography: Post-Sandy, First Surface (NJ) | 26 Oct to 5 Nov 2012 | 1.5 m | https://coast.noaa.gov/digitalcoast |
|  | 2014 NOAA Ortho-rectified Mosaic of Hurricane Sandy Coastal Impact Area | 1 Jan to 21 Apr 2014 | 0.35 m | https://coast.noaa.gov/digitalcoast |
|  | 2014 NOAA Post-Sandy Topobathymetric LiDAR: Void DEMs South Carolina to New York | Nov 2013 to Jun 2014 | 1 m | https://coast.noaa.gov/digitalcoast |
| Cedar Island | NAIP Digital Ortho Photo Image | 30 May 2011 | 1 m | https://gdg.sc.egov.usda.gov/ |
|  | 2010 VA Information Technologies Agency (VITA)/VA Geographic Information Network (VGIN) Lidar: Eastern Shore, VA (Accomack and Northampton Counties) | 21-28 Mar 2010 | 1 m | https://coast.noaa.gov/digitalcoast |
|  | 2013 VITA-VGIN VBMP 2013 Ortho Imagery SP South 12 inch | 14 Feb to 19 Apr 2013 | 0.30 m | http://vgin.maps.arcgis.com/home/index.html |
|  | 2012 USGS Topographic Lidar: Northeast Atlantic Coast Post-Hurricane Sandy | 5-29 Nov 2012 | 1 m | https://coast.noaa.gov/digitalcoast |
|  | 2014 NOAA Ortho-rectified Mosaic of Hurricane Sandy Coastal Impact Area | 01 Jan to 21 Apr 2014 | 0.35 m | https://coast.noaa.gov/digitalcoast |
|  | 2014 NOAA Post-Sandy Topobathymetric LiDAR: Void DEMs South Carolina to New York | Nov 2013 to Jun 2014 | 1 m | https://coast.noaa.gov/digitalcoast |
