# Supplementary material for: Using a Bayesian network to understand the importance of coastal storms and undeveloped landscapes for the creation and maintenance of early successional habitat
Source: PLoS One. 2019 Jul 25;14(7):e0209986. doi: 10.1371/journal.pone.0209986 (PMC6657824; doi:10.1371/journal.pone.0209986)
Supplement: S3 Table — Under this scale, a combination of landscape characteristics was considered ‘likely habitat’ if it was associated with a BN probability ≥ 0.66, ‘uncertain’ with a probability 0.33–0.66, and ‘unlikely habitat’ with a probability ≤ 0.33. We assumed that the IPCC scale accurately delineated habitat if the landscape characteristics associated with the majority of piping plover nest points had a probability ≥ 0.66 of being habitat and if the characteristics associated with the majority of random points had a probability ≤ 0.33 of being habitat. (DOCX) [file pone.0209986.s005.docx]

S3 Table. Effectiveness of the Intergovernmental Panel on Climate Change’s (IPCC’s) likelihood scale [8] for defining habitat predictions made by the Plover Habitat Bayesian network (BN). Under this scale, a combination of landscape characteristics was considered ‘likely habitat’ if it was associated with a BN probability > 0.66, ‘uncertain’ with a probability 0.33–0.66, and ‘unlikely habitat’ with a probability < 0.33. We assumed that the IPCC scale accurately delineated habitat if the landscape characteristics associated with the majority of piping plover nest points had a probability > 0.66 of being habitat and if the characteristics associated with the majority of random points had a probability < 0.33 of being habitat.

|  | **Nests** | **Random Points** |
| --- | --- | --- |
| Total # Points | 289 | 278 |
| # Points where *p* > 0.66 (%) | 281 (98%) | 26 (9%) |
| # Points where 0.33 < *p* > 0.66 (%)  [# Points where *p* = 0.5 (%)] | 8 (3%)  [2 (1%)] | 16 (6%)  [8 (3%)] |
| # Points where *p* < 0.33 (%) | 0 | 236 (85%) |
